# Identification and therapeutic investigation of biomarker genes underpinning hepatocellular carcinoma: an *in silico* study utilising molecular docking and dynamics simulation

**DOI:** 10.3389/fbinf.2025.1567748

**Published:** 2025-09-19

**Authors:** Jishnu Ghosh, Abdullah M. Alshahrani, Aritra Palodhi, Debarghya Bhattacharyya, Subhadip Das, Sunil Kanti Mondal, Abul Kalam, S. Rehan Ahmad, Chittabrata Mal

**Affiliations:** 1 Department of Biotechnology, University of Burdwan, Bardhaman, India; 2 Department of Basic Medical Science, College of Applied Medical Sciences, Khamis Mushait Campus, King Khalid University (KKU), Abha, Saudi Arabia; 3 Department of Bioinformatics, Maulana Abul Kalam Azad University of Technology, West Bengal, Haringhata, India; 4 Bidhannagar College, Kolkata, West Bengal, India; 5 Hiralal Mazumdar Memorial College for Women, West Bengal State University, Government of West Bengal, Kolkata, West Bengal, India

**Keywords:** differential gene identification, PPI network, biomarkers, docking, molecular dynamics simulation

## Abstract

**Background:**

Hepatocellular carcinoma (HCC) is the third leading cause of cancer-related mortality globally, and ranks fifth in terms of incidence. It primarily affects males and has a high prevalence in Asia. Risk factors include hepatitis B and C, liver cirrhosis, nonalcoholic fatty liver disease (NAFLD), and alcohol consumption. Late-stage diagnosis results in a poor survival rate of approximately 20%, underscoring the need for early detection methods to improve the survival rates. This study aimed to identify prognostic biomarkers for HCC through bioinformatic analysis of microarray datasets, providing insights into potential therapeutic targets.

**Methods:**

We analyzed five microarray datasets, comprising 402 HCC samples and 121 control samples. To identify relevant biological pathways, we conducted differential gene expression, Gene Ontology (GO), and KEGG pathway enrichment analyses. We identified hub genes and quantitatively assessed transcription factors and microRNAs targeting these genes. Additionally, molecular docking and dynamic simulations (100 ns) were used to identify potential drug candidates capable of inhibiting the activity of differentially expressed hub genes.

**Results:**

Our bioinformatic approach identified several promising HCC biomarkers. Among these, CDK1/CKS2 was identified as a key therapeutic target, with a regulatory role in HCC pathogenesis, suggesting its potential for further investigation. Digoxin (DB00390) has been highlighted as a potential repurposed drug candidate because of its favorable drug-likeness and stability, as confirmed by virtual screening, ADMET analysis, molecular docking study and dynamic simulations.

**Conclusion:**

This study enhances our understanding of HCC biology and offers new insights into drug interactions. It presents several promising biomarkers for the early diagnosis, prognosis, and therapy. Further investigation into CDK1/CKS2 as a therapeutic target and the role of the identified biomarkers could contribute to improved diagnostic and therapeutic strategies for HCC.

## Highlights

• Prognostic biomarkers for hepatocellular carcinoma (HCC) were identified by analyzing five microarray datasets containing 402 cancer and 121 control samples.
• CDK1 has been observed to be upregulated in HCC samples and is considered a potential oncogene involved in various cellular functions and processes.
• Molecular docking studies indicated that Digoxin (DB00390) has a strong binding affinity for the CDK1/CKS2 protein.
• Root mean square deviation (RMSD) analysis confirmed the stability of the complexes throughout the 100 ns production period.
• Molecular dynamics simulations revealed the flexibility of the binding site, the binding free energy of the complexes, and region-specific residue contributions to ligand binding.

## 1 Background

Liver cancer primarily comprises of hepatocellular carcinoma (HCC) (Bray et al., 2024). It ranks third in cancer-related mortality globally, and is the fifth most common cancer worldwide (Wang et al., 2021). The incidence of HCC is notably higher in males than in females (Chidambaranathan-Reghupaty et al., 2021; Singh et al., 2020), with most cases being reported in Asia (Marques et al., 2020). The risk factors for HCC are diverse, and include HBV or HCV infection, liver cirrhosis, nonalcoholic fatty liver disease (NAFLD), and alcoholism (Ghouri et al., 2017; Bray et al., 2024). HCC induced by HBV and HCV is believed to be particularly prevalent (Ghouri et al., 2017; Singh et al., 2020). Late-stage diagnosis of HCC significantly contributes to early mortality, with survival rates of approximately 20% in patients diagnosed at advanced stages (Zhou et al., 2020). Conversely, early detection can improve survival rates by nearly 70% (Tsuchiya et al., 2015). Numerous pathological biomarkers associated with HCC have been identified, many of which serve as prognostic indicators for poor outcomes. These biomarkers include genes and non-coding RNAs that regulate pathways controlling both proliferative and non-proliferative cellular activities, and may also function as immune checkpoint inhibitors (Marques et al., 2020).

Non-coding RNAs, including miRNAs, lncRNAs, and snoRNAs, play a significant role in cellular activities in hepatocellular carcinoma (HCC) by regulating oncogenes and associated signaling pathways (Arefnezhad et al., 2024). Identifying specific biomarkers for HCC is a critical area of research (Schlosser et al., 2022). Although numerous biomarkers have been implicated in the disease, microarray data analysis methods have become widely employed to predict causative biomarkers in various cancers, including HCC. Several studies have utilized bioinformatic approaches to investigate cancers (Shil et al., 2022; Mitra et al., 2023). These studies have adopted diverse methodologies to identify potential biomarkers and to examine their roles in disease prognosis. Most studies have focused on microarray datasets from the Gene Expression Omnibus (GEO) database to identify prognostic genes, miRNAs, and transcription factors associated with HCC.

Our study aimed to identify significant biomarkers that can act as prognostic indicators for hepatocellular carcinoma (HCC) through comprehensive bioinformatics analysis. We obtained five datasets from the Gene Expression Omnibus (GEO) database and performed differential gene expression analysis to identify common biomarkers between diseased and control samples. To further our investigation, we employed various bioinformatics tools for Gene Ontology (GO) and KEGG enrichment analyses, hub gene identification, and survival analysis. Additionally, we conducted a quantitative assessment of transcription factors (TFs) and miRNAs targeting the differentially expressed genes (DEGs). We also performed virtual screening and selected potential drug candidates based on ADMET analysis and found Digoxin (DB00390) as an inhibitor of CDK1. Digoxin, a purified digitalis preparation derived from the leaves of *Digitalis lanata* (foxglove), is primarily used in the therapy of congestive heart failure, breast cancer (Kurzeder et al., 2025) and prostate cancer (Lin et al., 2004). Recently, in hypoxia-induced mice models, HCC tumor size has been significantly reduced by administering Digoxin (Zhang M. S. et al., 2022). However, the detailed mechanism remains unknown. Following the identification of key biomarkers, we applied molecular docking and molecular dynamics simulation techniques to explore drug candidates that may inhibit gene activity, potentially hindering liver cancer progression and offering therapeutic options for patients with HCC. These *in silico* methods significantly reduce both the cost and time associated with drug discovery while ensuring a reasonable level of accuracy (Aghajani et al., 2022).

## 2 Methods

### 2.1 Collection of datasets

The microarray datasets GSE41804 (Hodo et al., 2013), GSE45267 (Wang et al., 2013), GSE62232 (Schulze et al., 2015), GSE112790 (Shimada et al., 2019), and GSE121248 (Wang et al., 2007) were obtained from the Gene Expression Omnibus repository (https://www.ncbi.nlm.nih.gov/geo/). Each dataset belonged to the platform GPL570 (HG-U133_Plus_2) Affymetrix Human Genome U133 Plus 2.0. GSE41804 consists of 20 resected live tumor tissues and resected non-tumor liver tissues. GSE45267 contained 48 primary HCC samples and 39 non-cancerous samples. GSE62232 contained 81 HCC solid tumors and 10 non-tumor liver tissues. GSE112790 contained 183 liver cancer tumor tissues and 15 normal liver tissues. GSE121248 contained 70 tumor samples and 37 adjacent normal samples.

### 2.2 Differential gene expression analysis

Differential gene expression analysis was conducted using GEO2R (https://www.ncbi.nlm.nih.gov/geo/geo2r/) for the GSE41804, GSE45267, GSE62232, GSE112790, and GSE121248 datasets. Statistically significant up- and downregulated genes were filtered based on an adjusted p-value (adj. P-value) < 0.05 and |Log2FC| > 2. To identify the common up- and downregulated genes, Venn diagrams were constructed using a Multiple List Comparator (https://molbiotools.com/listcompare.php).

### 2.3. Gene ontology (GO) and KEGG pathway enrichment analysis

GO and KEGG enrichment analyses were performed using DAVID (DAVID Functional Annotation Tools ncifcrf.gov). The groups of common up-and downregulated genes were analyzed separately. An adjusted P-value <0.05 was considered to identify statistically significant terms and pathways.

### 2.4 Construction of PPI network and hub gene selection

The protein-protein interaction network of the common up-and downregulated gene set was generated in the Integrated Interactions Database (IID utoronto.ca) and by selecting “Retrieve all PPIs of query proteins’ in the option ‘Select retrieval strategy.” Network analyses were performed using Cytoscape (https://cytoscape.org/) to identify the top 10 hub genes using the plugin Cytohubba (through the EPC algorithm).

### 2.5 Identification of transcription factors (TF) associated the DEGs

The TF2DNA database (https://www.fiserlab.org/tf2dna_db/) was used to identify the Transcription factors (TFs) that regulate the transcription of upregulated and downregulated genes. The species chosen was *Homo sapiens*, Sources: TF2DNA (computational), and p-value = 0.0001.

### 2.6 Identification of miRNA targets

The miRWalk database (http://mirwalk.umm.uni-heidelberg.de/) was used to identify the miRNAs that target the mRNAs of the upregulated and downregulated genes. The top five upregulated and downregulated genes were chosen for analysis.

### 2.7 Survival analysis and validation of hub genes

GEPIA (http://gepia.cancer-pku.cn/) was used to identify potential candidate biomarkers associated with liver Hepatocellular Carcinoma (LIHC) prognosis. For this purpose, we assessed the genes that showed statistically significant (p < 0.05) Overall Survival (OS) and Disease-Free survival of patients (DFS) affected with LIHC. The survival analysis was done using the “overall survival” method and the median group cutoff was set to 50% for both high and low and the hazards ratio was taken into consideration. The expression levels of these genes in LIHC-affected tissues were determined by comparing their expression levels in normal tissues. Analyses were based on the TCGA-LIHC cohort.

### 2.8 Virtual screening and molecular docking

The necessary PDB file (PDB ID: 6GU7) was downloaded from the Protein Data Bank. This file contains the crystalized structure of CDK1, CDK1 regulatory subunit 2 and the co-crystallized molecule AZD-5438 (Wood et al., 2019). AZD-5438 is identified as a potential inhibitor of CDK1/CKS2 (Byth et al., 2009). CDK1 shows high co-occurrence and co-expression with CKS2 (Zhang et al., 2019). The protein file was prepared by removing the water molecules and other ligand molecules, adding missing hydrogens and fixing the atom names in the Discovery Studio (2024) prior to virtual screening. The modified PDB file was submitted to the Drugrep (cao.labshare.cn) server for virtual screening using the databases of FDA approved drugs (contains 4714 drugs) and experimental drugs (contains 6883 drugs) (Gan et al., 2023) out of the three available drug libraries (FDA approved drug library, experimental drug library and traditional Chinese medicine). After screening, DB00390, DB00511, DB00696, DB00872, DB01396, DB09102, and DB11581 were selected for repurposing based on their binding affinity with CDK1/CKS2. These ligands were docked (blind docking) in the HDOCK server (http://hdock.phys.hust.edu.cn/) and used for further analysis. Additionally, AZD-5438 was docked with the CDK1/CKS2 to obtain its binding affinity and root mean square deviation (RMSD) for comparison.

### 2.9 ADMET analysis

Properties, such as absorption, distribution, metabolism, excretion, and toxicity, were tested for the selected molecules to determine their competence as drug molecules. ADMET analysis was performed using the ADMETlab 3.0 web server (Fu et al., 2024). For the calculation of LD50, ProTox web server (Banerjee et al., 2024) was used. Drug likeness was calculated using the pkCSM web server (Pires et al., 2015).

### 2.10 Molecular dynamics simulation

Necessary molecular dynamics simulations were performed to concretize the results of the molecular docking study. MD simulations were performed using Gromacs 2019 software and the Compute Unified Device Architecture (CUDA) API on NVIDIA graphics processors. CDK1/CKS2 and CDK1/CKS2-Digoxin complex derived from molecular docking were imported into the gromacs and converted from *.pdb to *.gro format. Gromacs works with proteins, nucleic acids, and lipids, with predefined force field parameters available in the software itself. However, these required force field parameters are not available for the ligands in Gromacs. So, a third-party server, “Ligand parameter generator” or “Ligpargen” (Jorgensen and Tirado-Rives, 2005; Dodda et al., 2017a; Dodda et al., 2017b) was used to generate force field parameters for ligands. The output files from Ligpargen are used directly in gromacs, which define its topology and force-field parameters. The protein-ligand complexes were placed in a virtual 3D cube filled with water and simulated using the SPC/E water model. The entire system was then electrically neutralized by adding the necessary monovalent Na+ and Cl-ions, and a GROningen MOlecular Simulation (GROMOS) field was applied. A constant temperature (Akharume and Adedeji, 2023) of 300 K and 1.00 bar pressure was applied to the system using NPT and NVT as an ensemble class. A leapfrog integrator was used to compile the motion equations with a 2-fs time step size. A Verlet (buffered neighbor searching) cutoff scheme was used for non-bonded interactions, a modified Berendsen thermostat was applied to control the temperature, and the Parrinello-Rahman method was used to regulate pressure. Particle Mesh Ewald was used for calculations of long-range electrostatics. Both the short-range electrostatic and short-range Van der Waals cutoffs (Piana et al., 2012) were set to 1.0 nm. Standard Gromacs protocols were used to minimize the energy of the system and perform the simulation for 100 ns. Similar parameters were used to simulate the apoprotein (CDK1/CKS2) for 100 ns for comparison. Tools provided by Gromacs software were used to calculate the trajectories of individual atoms. The same software was used to plot the Root Mean Square Deviation (RMSD), Root Mean Square Fluctuation (RMSF), solvent-accessible surface area (SASA), Radius of Gyration (RoG) for both the CDK1/CKS2 and CDK1/CKS2-Digoxin complex. SASA was calculated using the rolling ball algorithm (Richmond, 1984). Additionally, the number of hydrogen bonds, hydrogen bond angle and bond distance during simulation time (100 ns) were calculated for CDK1/CKS2-Digoxin complex. Another tool g_mmpbsa (Kumari et al., 2014) was used to calculate the Molecular Mechanics - Poisson-Boltzmann Surface Area or MM-PBSA from the topology and trajectory data of CDK1/CKS2-Digoxin complex generated from the simulation.

## 3 Results

### 3.1 Common set of genes differentially expressed across the datasets

**FIGURE 1:**
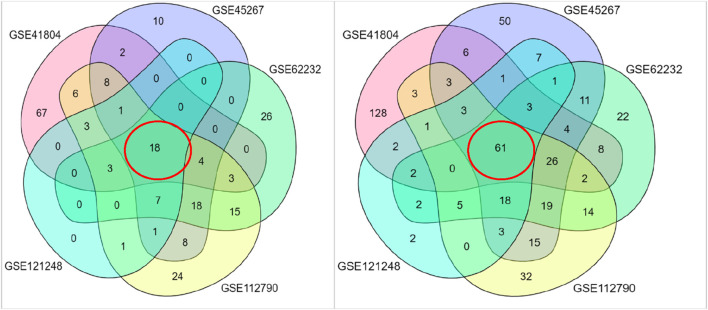
Venn diagrams of differential gene expression analysis across five microarray datasets. Common up and downregulated genes are circled in red.

Differential gene expression analysis was performed on five datasets (GSE41804, GSE45267, GSE62232, GSE112790, and GSE121248) using the cut-off criteria of an adj. P-value <0.05 and |log2FC|> 2. Using the Venn diagram (Figure 1), we identified 19 upregulated and 61 downregulated genes that were common among these datasets.

### 3.2 DEG enriched GO terms and KEGG pathways

From the GO and KEGG pathway enrichment analysis in DAVID, 18 GO terms and one pathway were enriched by the upregulated genes, whereas 54 GO terms and 9 KEGG pathways were enriched by the downregulated genes. The GO terms were Biological Process (BP), Cellular Components (CC), and Molecular Functions (MF). The results are shown in Supplementary Table S1 *.*

### 3.3 PPI network revealed hub genes

The PPI network of both up-and downregulated genes constructed in the Integrated Interactions Database (IID) was analyzed in Cytoscape using the plugin Cytohubba (EPC algorithm). We obtained the top 10 nodes among the upregulated and downregulated genes, as scored by Cytohubba. Since CDK1 and ESR1 in the up-and downregulated networks were the highest ranked, they were predicted to be hub genes.

### 3.4 Transcription factors (TF) targeting the top DEGs

The TFs of the top five upregulated and downregulated genes were identified using the TF2DNA database. The upregulated genes, NEK2, CDK1, SULT1C2, RRM2, and ANLN, were regulated by 22, 76, 7, 42, and 40 TFs, respectively. Similarly, the downregulated genes, ESR1, LIFR, CXCL12, ADRA1A, and IGF2, were regulated by 113, 30, 36, 11, and 136 TFs, respectively. The results are presented in Supplementary Table S2.

### 3.6 Top DEGs targeted by miRNA

The top five upregulated and downregulated genes were subjected to analysis in the miRWalk database to identify miRNAs that participate in the post-transcriptional modification of these genes. The upregulated genes NEK2, CDK1, SULT1C2, RRM2, and ANLN were targeted by 12, 14, 10, 8, and 7 miRNAs, respectively. Similarly, the downregulated genes, ESR1, LIFR, CXCL12, ADRA1A, and IGF2 were targeted by 58, 36, 36, 32, and 31 miRNAs, respectively. The results are presented in Supplementary Table S3.

### 3.7 Patient survivability affected due to hub gene expression

**FIGURE 2:**
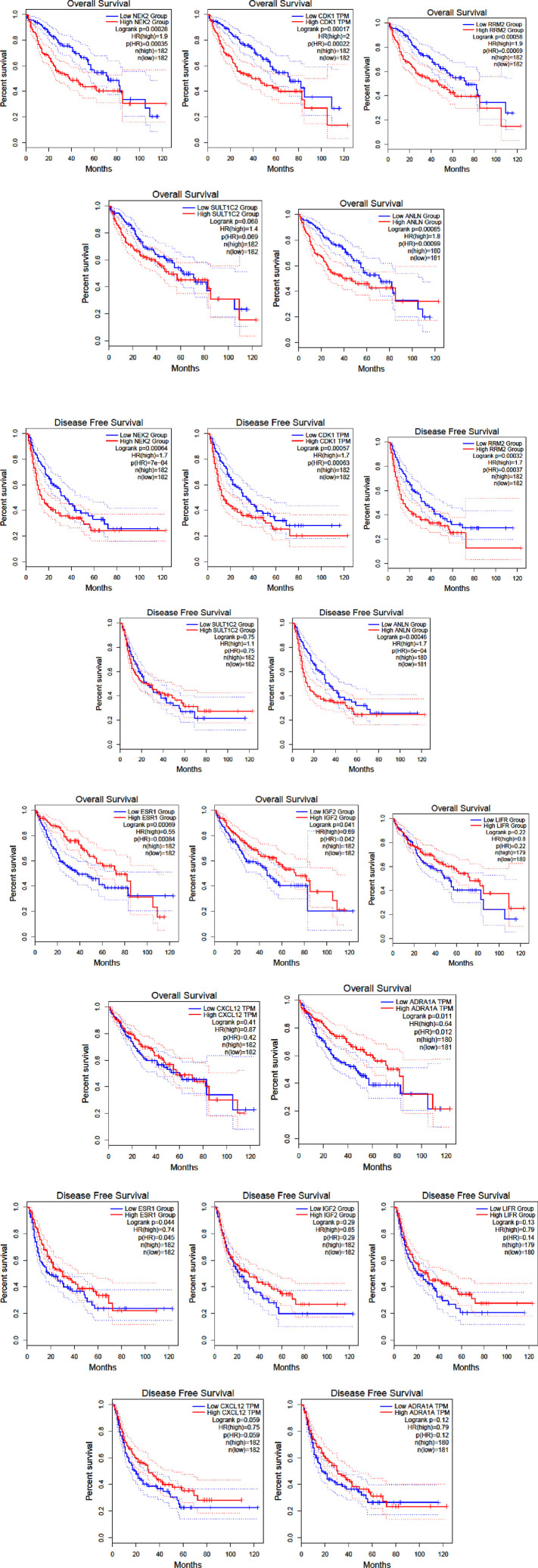
Overall survival and Disease free survival of the top 5 up and downregulated genes in HCC.

The top five upregulated and downregulated genes were analyzed for their association with overall and disease-free survival in LIHC using the TCGA-LIHC cohort, which was divided into high and low expression groups. Kaplan-Meier analysis of the upregulated genes NEK2, CDK1, SULT1C2, RRM2, and ANLN showed that the CDK1 high-expression group had significantly poorer OS and DFS than the low-expression group and all other upregulated gene groups (Figure 2). In contrast, among the downregulated genes ESR1, LIFR, CXCL12, ADRA1A, and IGF2, ESR1 had the poorest OS and DFS in the low-expression group of LIHC patients compared to the other downregulated gene groups (Figure 2).

**FIGURE 3:**
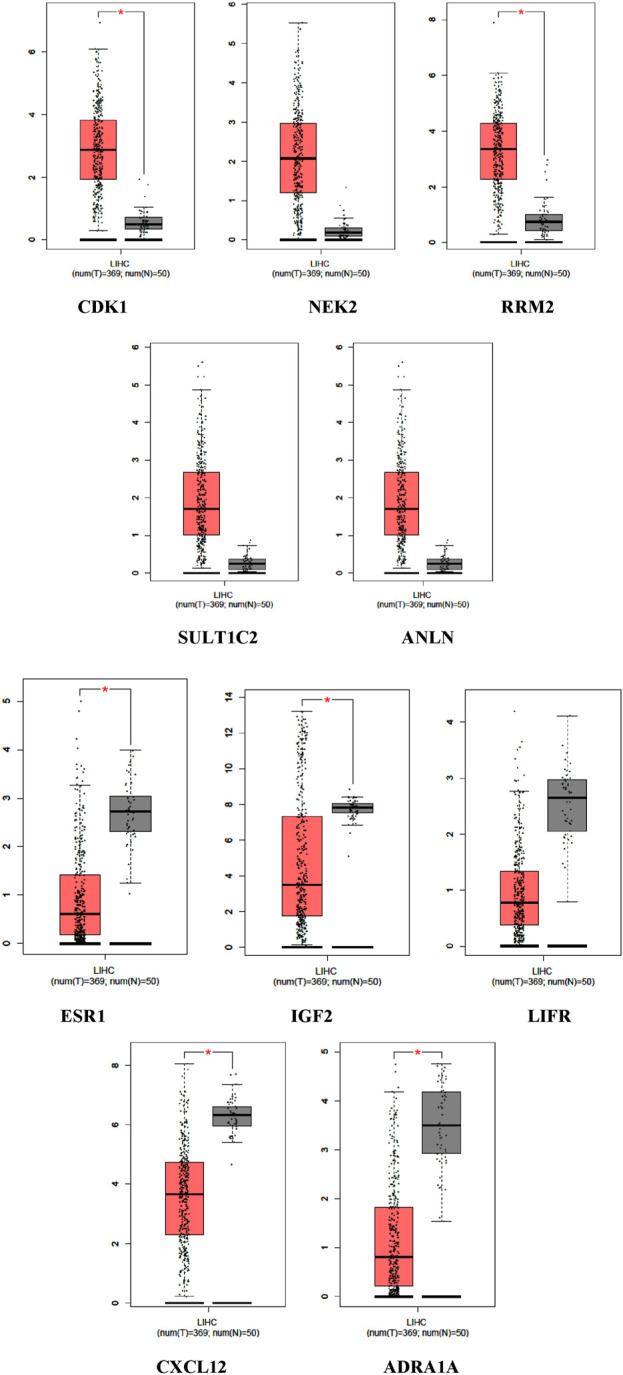
Expression level of the up and downregulated genes in log_2_ (TPM + 1) in LIHC patients compared to normal.

The expression levels of the upregulated and downregulated genes in LIHC-affected and normal TCGA cohort data were also evaluated using GEPIA (Figure 3). All genes, except NEK2 and ANLN, showed statistically significant upregulation in LIHC samples compared to normal samples. Similarly, except for LIFR, all other genes showed statistically significant downregulation in LIHC samples compared to that in normal samples.

### 3.8 Properties of drug molecules identified by ADMET analysis

ADMET analysis provides insight into various properties of drug molecules, such as absorption, distribution, metabolism, excretion, and toxicity (Tables 1–5). These properties help determine whether the drug should be taken orally, its absorption rate in the gastrointestinal tract, the likelihood of being metabolized by the cytochrome P450 group of enzymes, and the excretion rate of the drug molecule (Zhao et al., 2021). It also sheds light on potential toxic effects, such as hERG-inhibition, skin sensitization, hepatotoxicity, neurotoxicity, carcinogenicity, and various effects on the eyes.

**TABLE 1:** Absorption properties of the drug candidates.

Absorption	DB00390	DB00511	DB00696	DB00872	DB01396	DB09102	DB11581
Caco-2 Permeability	−4.974	−5.21	−5.029	−4.931	−5.192	−5.002	−4.927
MDCK Permeability (10–6 cm/s)	−4.892	−4.773	−4.748	−4.685	−4.763	−4.618	−4.734
PAMPA (logPeff)	0.26	0.411	0.108	0.017	0.954	0.574	0.811
Pgp-inhibitor	0.001	0.001	0.095	0.985	0.001	0.988	0.021
Pgp-substrate	0.933	0.998	1.0	0.812	1.0	0.306	0.058
HIA	0.0	0.0	0.019	0.0	0.005	0.001	0.0

**TABLE 2:** Distribution properties of the drug candidates.

Distribution	DB00390	DB00511	DB00696	DB00872	DB01396	DB09102	DB11581
PPB (%)	91.751	95.179	94.243	98.012	95.487	98.201	97.635
VDss (L/kg)	−0.393	−0.373	−0.287	−0.032	−0.395	0.076	0.114
BBB	0.977	0.043	0.85	0.809	0.019	0.0	0.598
OATP1B1 inhibitor	0.913	0.926	0.08	0.79	0.756	0.996	0.885
OATP1B3 inhibitor	0.0	0.001	0.007	0.39	0.069	0.994	0.69
BCRP inhibitor	0.0	0.0	0.001	0.064	0.00	0.269	0.0
MRP1 inhibitor	1.0	0.993	0.693	0.887	0.877	0.948	0.293

**TABLE 3:** Metabolic properties of the drug candidates.

Metabolism	DB00390	DB00511	DB00696	DB00872	DB01396	DB09102	DB11581
CYP2D6 substrate	yes	yes	yes	no	yes	yes	yes
CYP3A4 substrate	yes	yes	no	yes	yes	yes	yes
CYP1A2 inhibitor	yes	yes	no	no	yes	yes	no
CYP2C19 inhibitor	no	no	no	no	no	no	yes
CYP2C9 inhibitor	yes	yes	no	no	yes	yes	no
CYP2D6 inhibitor	yes	yes	yes	no	yes	yes	yes
CYP3A4 inhibitor	yes	no	no	no	no	yes	yes

**TABLE 4:** Excretion properties of the drug candidates.

Excretion	DB00390	DB00511	DB00696	DB00872	DB01396	DB09102	DB11581
CL_plasma_ (mL/min/kg)	3.56	2.136	7.853	4.619	1.705	3.578	1.003
T_1/2_ (hours)	2.583	2.053	0.946	0.506	2.339	1.103	1.355

**TABLE 5:** Toxicity properties of the drug candidates.

Toxicity	DB00390	DB00511	DB00696	DB00872	DB01396	DB09102	DB11581
hERG Blockers	no	no	no	no	no	yes	no
hERG Blockers (10um)	no	no	no	yes	no	yes	no
skin sensitization	yes	yes	yes	yes	yes	yes	yes
AMES Mutagenicity	yes	yes	yes	yes	yes	yes	yes
Carcinogenicity	no	yes	yes	yes	yes	yes	yes
Eye Corrosion	no	no	no	no	no	no	no
Eye Irritation	no	no	no	yes	no	no	no
Human Hepatotoxicity	yes	yes	yes	yes	yes	yes	yes
Drug-induced Neurotoxicity	no	yes	yes	yes	yes	yes	yes
LD50 (mg/kg)	5105	5105	800	3550	650	700	2000

Caco-2 is a monolayer cell line that is used as a model for the emulation of intestinal drug absorption (Van Breemen and Li, 2005). A high value (>−5.15 log unit) indicates that the drug is likely to be absorbed in the intestines upon oral administration (Flores‐Holguín et al., 2021). DB00390, DB00872, and DB11581 have high permeability, suggesting that these drugs have higher absorption rates than the rest. All drugs except DB00872 and DB09102 can act as p-glycoprotein inhibitors (pgp-inhibitors), while only DB11581 can act as a pgp-substrate. This has a significant effect on drug fate.

The value of the volume of distribution (VDss) shows whether the drug is likely to be distributed in the plasma or tissue. Higher VDss values (>−0.45) suggest that the drug is distributed in the tissue (Han et al., 2019). All drugs, except DB09102 and DB11581, were estimated to be distributed in the plasma. DB00390, DB00696, DB00872, and DB11581 are more likely to cross the blood-brain barrier (Pires et al., 2015).

Drugs are typically metabolized by Cytochrome P450 enzymes including CYP3A4, CYP1A2, CYP2C1, CYP2C9, and CYP2D6 (Ogu and Maxa, 2000). It is important for the drug to be metabolized by cytochrome P450 enzymes, as this phenomenon prohibits the accumulation of the drug in the system and causes side effects (Jindal et al., 2019). DB09102 and DB00390 have a high chance of being metabolized, whereas DB00696 and DB00872 are likely to accumulate in the body.

The CL_plasma_ parameter shows the renal absorption rate of a drug (Horde and Gupta, 2024). DB00390, DB00511, DB00872, and DB09102 exhibited low renal clearance and DB01396 as well as DB11581 exhibited extremely low renal clearance. These drugs can have high efficiency as they are more likely to be reabsorbed by the kidneys. However, the T_1/2_ values, which indicate the half-life of the drug inside the body, were favorable for DB00390 and DB00511.

While assessing toxicity, it was found that except DB00390, all the other drugs exhibited some levels of carcinogenicity. None of the drugs caused eye corrosion, and only DB00872 caused eye irritation. However, all drugs displayed some levels of skin sensitization, Mutagenicity, and Hepatotoxicity. DB00390 alone showed no drug-induced neurotoxicity. The lethal Dose 50 (LD50) parameter was used to show the amount of drug needed to be lethal to 50% of the test subjects. This value was calculated in mg/kg units. DB00390 and DB00511 are safe for oral administration (Gadaleta et al., 2019).

All the drugs were screened to estimate their drug-likeness based on their physicochemical characteristics (Table 6). DB00696 and DB00872 fall within the range of 100–600 g/mol and satisfy rule-1 however all the other drugs slightly deviate from this range and have molecular weights of approximately 700 g/mol. DB00872 only follows rule 2 and shows a log P value within the 0–3 log mol/L range. All drugs chosen here show 0-11 rotatable bonds and follow rule 3. DB00390, DB00696, and DB00872 show hydrogen bond acceptors less than 12, maintaining rule 4. All drugs showed less than seven hydrogen bond donors, upholding rule 5. However, no drug falls within the range of 0–140 A^2^ surface area and does not uphold rule 6 (Alsfouk et al., 2024). The six drug selection rules applied in this study should be regarded as flexible guidelines rather than rigid cutoffs, similar to the Rule of Five (RoF), which suggests that compounds failing two or more criteria may have poor oral bioavailability, though each criterion is often used individually as a filter (Yusof and Segall, 2013). Moreover, DB00390 (Digoxin) is a well-established drug molecule, and digitalis-based drugs such as digitoxin and Digoxin are still in clinical use as oral medications for treating heart failure and atrial arrhythmias (Zhou et al., 2023).

**TABLE 6:** Physicochemical properties to determine drug-likeness.

Name	Molecular weight (g/mol)	LogP	Rotatable bonds	H-bond acceptors	H-bond donors	Surface area (A^2^)
DB00390	757.765	4.9059	3	11	2	315.675
DB00511	774.731	5.6738	6	14	2	323.322
DB00696	569.577	2.506	1	9	0	244.996
DB00872	496.570	4.86952	3	4	2	219.165
DB01396	732.694	5.3213	6	13	4	305.916
DB09102	722.762	5.6664	5	14	0	308.777
DB11581	852.329	9.6965	9	13	2	355.938

### 3.9 Candidate drugs selected by molecular docking analysis

**FIGURE 4:**
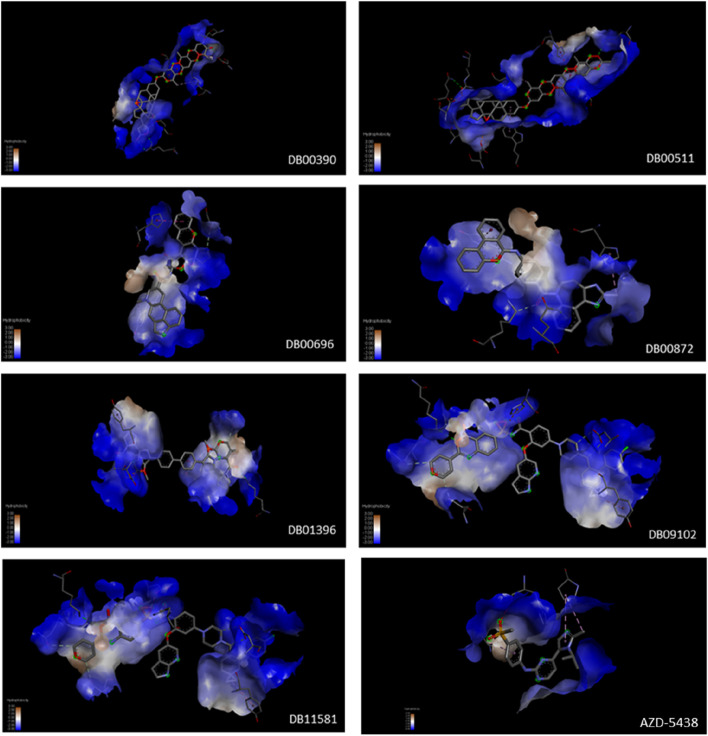
Hydrophobic interactions of DB00390, DB00511, DB00696, DB00872, DB01396, DB09102, DB11581 and AZD-5438 with CDK1/CKS2 receptor. Hydrophobicity varies from +3 (brown) to −3 (blue).

Seven drug candidates were selected for molecular docking studies: DB00390, DB00511, DB00696, DB00872, DB01396, DB09102, and DB11581. The HDOCK server was used for the molecular docking study, and the results are summarized in Table 7. Four of the seven selected drugs, DB00390, DB00511, DB09102, and DB11581, showed high affinity for the receptor molecule. However, DB00511 had a high ligand RMSD, indicating that the drug is less likely to bind to the receptor compared to the other three drugs. DB00390 and DB00511 form two hydrogen bonds with the receptor, making the complexes more stable than the rest. DB09102 and DB11581 did not form any hydrogen bonds. DB00390 and DB00511 showed a high number of hydrophobic interactions (17 and 14, respectively). The remaining candidates showed a moderate number of hydrophobic interactions (Figures 4, 6). Although DB00511, DB09102, and DBB11581 showed high affinity, low ligand RMSD, and a considerable number of interactions, they were not considered because of their potential carcinogenicity and high toxicity values obtained from the previous ADMET analysis. Therefore, DB00390 was considered suitable for further molecular dynamic simulation studies.

**TABLE 7:** Docking score with CDK1/CKS2, RMSD and number of interacting bonds of DB00390, DB00511, DB00696, DB00872, DB01396, DB09102, DB11581.

Drug ID	Docking score	Ligand RMSD (Å)	Number of H-bonds	Number of hydrophobic interactions
DB00390	−245.01	50.77	2	17
DB00511	−245.66	64.29	2	14
DB00696	−201.7	65.35	1	10
DB00872	−177.17	62.12	1	10
DB01396	−222.61	43.71	1	8
DB09102	−247.75	43.46	0	12
DB11581	−247.75	43.46	0	12
AZD-5438	−129.94	64.62	0	8

**FIGURE 5:**
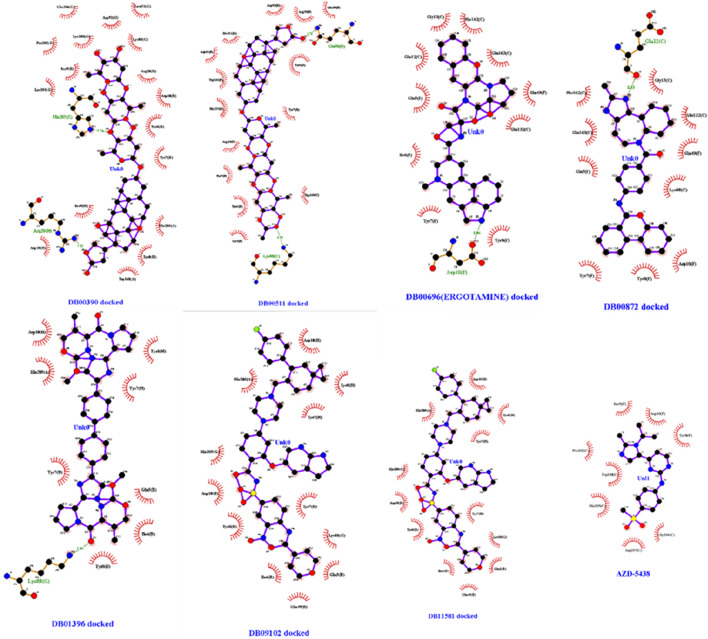
Hydrophobic interactions and hydrogen bonds of DB00390, DB00511, DB00696, DB00872, DB01396, DB09102, DB11581 and AZD-5438 with CDK1/CKS2 receptor (Ligplot analysis).

Moreover, a comparative study with a well known molecule (AZD-5438) was performed through successive molecular interactions to establish repurposed drug DB00390 as a potential inhibitor of CDK1/CKS2. While AZD-5438 showed nearly half affinity towards the CDK1/CKS2 complex compared to DB00390, the RMSD value of AZD-5438 was slightly less than DB00390. Furthermore, Ligplus analysis showed that the number of hydrogen bonds and hydrophobic interactions with CDK1/CKS2 dropped drastically (Figure 5) for AZD-5438, inferring Digoxin (DB00390) as a better inhibitor.

### 3.10 Validated receptor-drug interaction confirmed by molecular dynamics simulation

**FIGURE 6:**
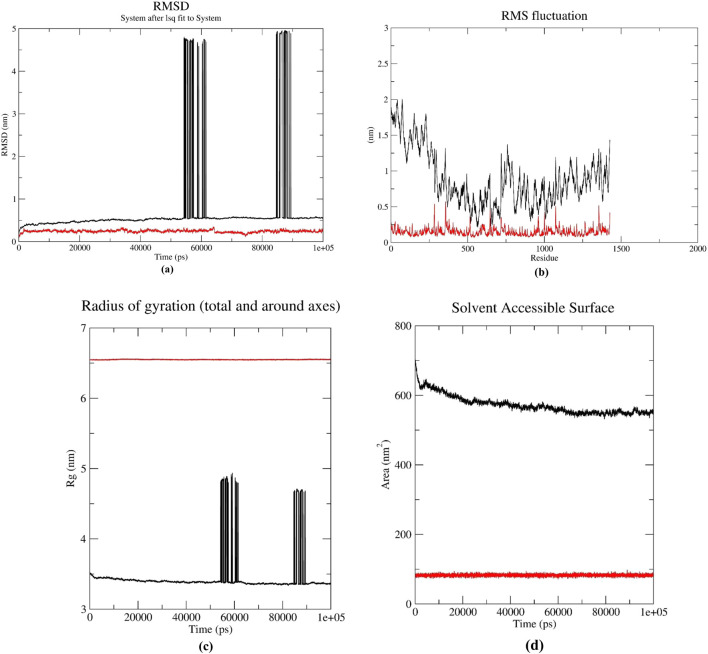
**(a)** RMSD, **(b)** RMSF comparison of CDK1/CKS2 (black) and CDK1/CKS2-Digoxin complex (red) **(c)** Radius of Gyration, **(d)** Solvent Accessible Surface Area.

**FIGURE 7:**
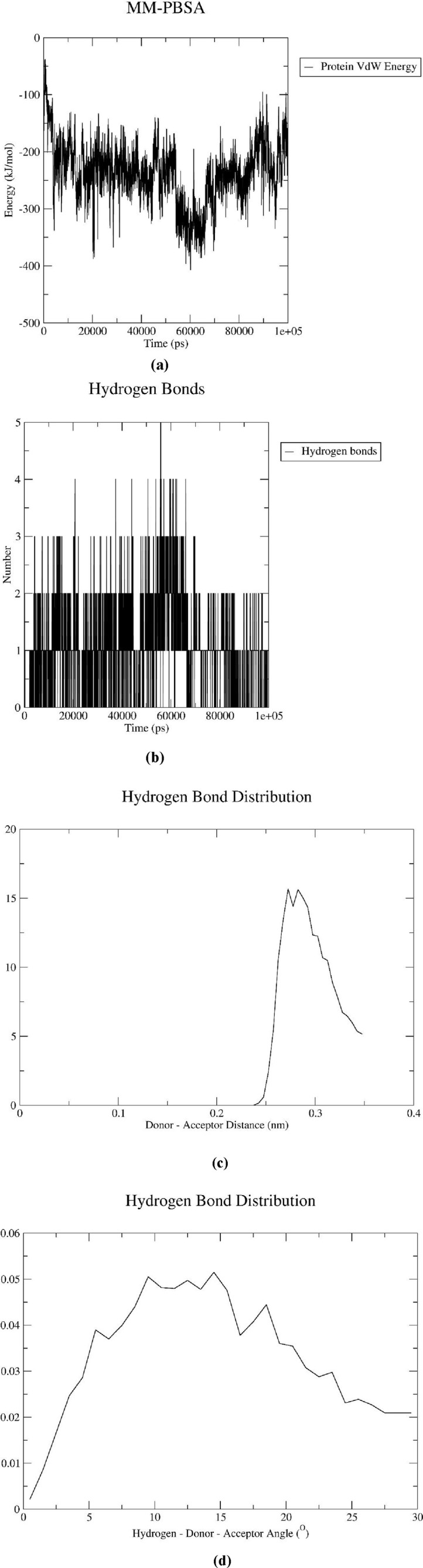
**(a)** MM-PBSA graph representing the total binding energy of the CDK1/CKS2-DB00390 complex, **(b)** Number of Hydrogen bonds, **(c)** Distribution of Hydrogen bond distance and **(d)** Distribution of Hydrogen bond angle during the 100 ns simulation.

Based on molecular docking and ADMET analyses, DB00390 (Digoxin) was used for the molecular dynamic simulation study. Various graphs i.e., RMSD, RMSF, RoG, SASA, MM-PBSA, Hydrogen bond number, angle and distance were derived from the molecular dynamics simulation results (Figure 6). Fluctuation of RMSD around 0.25 nm and decreasing to 0.1 nm at 70–80 ns indicates that the CDK1/CKS2-Digoxin complex is fairly stable (Maruyama et al., 2023). Comparatively, the RMSD of the apoprotein remained at 0.5 nm with occasional spikes reaching up to 4.5–5.0 nm, reflecting instability in the absence of the ligand (Figure 6a). This implies that compared to CDK1/CKS2, the CDK1/CKS2-Digoxin complex is more stable and has enhanced inhibitory potential and potentially more biologically effective. The average RMSF of each residue was calculated to determine the residues that fluctuated during the simulation. The residues of CDK1/CKS2 generally showed higher fluctuation compared to the CDK1/CKS2-Digoxin complex implying higher instability (Figure 6b). The radius of gyration (RoG) of CDK1/CKS2-Digoxin also showed low fluctuation around 5 nm. The RoG of CDK1/CKS2 stayed around 3.5 nm with two abrupt increases upto 5 nm and 4.7 nm at 60 ns and 85 ns respectively (Figure 6c). The solvent-accessible surface area is also a good indicator of protein complex compactness and stability. In our case, the SASA of CDK1/CKS2-Digoxin was fluctuating between 75 and 95 square nm compared to 600 square nm of CDK1/CKS2 (Figure 6d), which indicates that the protein remained compact and stable during the simulation (Bagewadi et al., 2023). Higher value of RoG but lower values of RMSD, RMSF and SASA suggest possible shift in mass distribution rather than instability of the CDK1/CKS2-Digoxin complex (Miu et al., 2008). The MM-PBSA graph shows that the total binding free energy drops from initial −50 kJ/mol to −400 kJ/mol at 40 ns and 60 ns and stays around −200 kJ/mol at the end of the simulation (Figure 7a). This gradual reduction of the total binding free energy indicates that the whole complex achieves stability over time (Kumari et al., 2014). Molecular dynamic simulation analysis shows 1–3 hydrogen bonds being generated between the protein and the drug during the simulation time period of 100 ns (Figure 7b). Hydrogen bond distances and angles show the fluctuation between 0.24 and 0.35 nm and 2–30° respectively, which infer a stable bonding between the protein and the drug (Figures 7c,d).

## 4 Discussion

Hepatocellular carcinoma (HCC) is one of the most challenging cancers to treat owing to its late diagnosis and complex characteristics. It often affects males more than females. Numerous studies have focused on bioinformatic analyses to identify biomarkers for HCC. In our study, we conducted a differential gene expression analysis across five microarray datasets, identifying 19 upregulated and 64 downregulated genes that were commonly observed in all datasets. These commonly upregulated and downregulated genes were then subjected to GO and pathway enrichment analyses. The number of significant GO terms and KEGG pathways enriched with the upregulated genes was significantly lower than that of their downregulated counterparts. Following this, we constructed a PPI network for the up-and downregulated gene sets of HCC and identified key hub genes through analysis of each of the gene sets. We selected CDK1 and ESR1 as hub genes for our analysis. From the differentially expressed gene sets, we selected the top five upregulated (CDK1, NEK2, RRM2, SULT1C2, and ANLN) and downregulated genes (ESR1, IGF2, LIFR, CXCL12, and ADRA1A) for our studies related to gene-miRNA and TF gene targets, including patient survival analysis. We found that these genes including CDK1 and ESR1 were targets of a large number of TFs and miRNAs. In addition, CDK1 and ESR1 showed significant and poor OS and DFS in patients with HCC, and they were validated to be upregulated and downregulated in patients with HCC. KEGG/GO enrichment analysis showed that various pathways can potentially be influenced by the upregulation of CDK1 gene, e.g., mitochondrial ATP synthesis coupled electron transport, G2/M transition of mitosis, protein serine/threonine kinase activity, *etc.* We found that ESR1 is involved in proteoglycan mediated cell signaling pathways and shaping the tumor microenvironment. This finding is supported by earlier research of Dituri et al. (2022). SOX transcription factors (SOX4, SOX7, SOX15, SOX17) and FOXM1 downregulated the expression of ESR1 (Xiao et al., 2019) while FOXN2 and FOXS1 were involved in upregulation of CDK1 expression (Liu et al., 2021).

The gene ESR1 encodes a transcription factor that is located in the chromosomal region chromosomal region 6q25.1 (Tsiambas et al., 2011). Its protein structure comprises N- and C-terminal domains, activation and ligand-binding domains, and DNA-binding domains. ESR1 has been proposed to play a regulatory role in liver, prostate, endometrial, and lung cancer (Hu et al., 2022). A cell line-based study found that ESR1 induces upregulation of lncRNA MEG3 under high glucose conditions, which hinders HCC progression (Cheng et al., 2022). A study found that miR-9-5p downregulates ESR1 gene activity in HCC, causing cancer cell proliferation, migration, and invasion (Wang et al., 2021). ESR1 can also regulate the expression of MMAA, an obesity-metabolism differential gene, to prevent HCC, as shown in a study in females (Zhang Y. et al., 2022).

CDK1 is a member of the serine-threonine protein kinase family, which is important during transitions from the G1/S or G2/M phase of the cell cycle (Enserink and Kolodner, 2010). Studies have shown that the gene activity of CDK1 is regulated by miR-582-5p in pathways involving CDK1 and AKT3, which in turn induces cell cycle arrest in the G0/G1 phase. miR-582-5p is downregulated (Zhang et al., 2015). CDK1 also plays a role in immune infiltration in HCC (Zou et al., 2020). A bioinformatic study revealed that CDK1, FOXM1, TCF7L1, E2F4, and SIN3A can act as important transcriptional regulators in HBV-induced HCC (Wang et al., 2018). Thus, CDK1 may act as a prognostic biomarker for HBV-induced HCC.

We propose that CDK1 and ESR1, identified as upregulated and downregulated genes, respectively, could serve as potential biomarkers for HCC. However, we focused exclusively on the upregulated gene CDK1 as a candidate for molecular docking and dynamics simulations, hypothesizing that a suitable drug could effectively inhibit CDK1 protein. In cancer, downregulated genes - particularly tumor suppressors-are typically not targeted for inhibition; rather, therapeutic strategies aim to restore or enhance their expression and function. In some contexts, increasing the activity of such genes may involve inhibiting their negative regulators instead. ESR1 (estrogen receptor 1) exhibits tumor-suppressive behavior in liver cancer, where its downregulation is associated with disease progression (Hishida et al., 2013). Studies have shown that ESR1 knockout in mouse models increases susceptibility to liver tumorigenesis (O’Brien et al., 2021), while its activation through estrogen signaling suppresses tumor growth (Fuentes and Silveyra, 2019). Given this tumor-suppressive role, ESR1 was excluded from further analysis focused on drug inhibition effects.

Other bioinformatic studies on HCC have identified CDK1 and ESR1 as potential hub genes (Table 8). For instance, Zhang et al. (2021) found that these genes were upregulated and downregulated, respectively, in HCC using a different set of microarray samples than those used in our study. Another study highlighted CDK1 and ESR1 as hub genes, both of which are associated with poor survival outcomes (Ni et al., 2019). In spite of some earlier research findings showing CDK1 as a prominent regulator of liver cancer (Ni et al., 2019; Sun and Zhang, 2020; Hao et al., 2021; Su et al., 2023), molecular docking studies have identified treatment options such the Phellodendron and Anemarrhena drug pair (PADP) as a promising inhibitor of CDK1 (Ruan et al., 2022). However, molecular dynamics modelling has not yet shown Digoxin and CDK1’s interaction behaviour. To bridge this gap, further evaluation of Digoxin’s mechanism of action inside cancer cells is necessary to understand its potential as a CDK1 inhibitor.

**TABLE 8:** A brief review of previous studies in connection to our study.

References	Dataset used	Key methodology	Status of CDK1 and ESR1 in the study
Ni et al. (2019)	GSE27635, GSE28248	Differential gene expression analysis, PPI network analysis, Hub gene selection and survival analysis, experimental validation	Six genes identified as hub genes including CDK1 in HCC.
Sun and Zhang (2020)	GSE45436	Differential gene expression, Survival analysis, Validation	CDK1 and ESR1 were among the significant Differentially expressed genes. However, they were not considered significant gene based on further analysis
Hao et al. (2021)	GSE62232 GSE40744	Differential gene expression analysis, Validation of hub gene expression, Construction of miRNA-mRNA regulatory network	CDK1 and ESR1 were predicted as hub genes for HCV related HCC and were found to be regulated by miR-122-5p and miR-221-3p
Zhang et al. (2021)	GSE14520, GSE25097, GSE36376, GSE57957, GSE76427, GSE121248	Differential Gene expression analysis, PPI network analysis, survival analysis, hub gene identification	88 upregulated and 40 downregulated genes were identified as hub genes, including CDK1 and ESR1 in HCC.
Ruan et al. (2022)	GSE62232	Differential gene expression analysis, Acquisition of Chemical Components and Targets of PADP, Construction of Traditional Chinese Medicine Compound Regulation Network, Molecular Docking Verification of Core Compounds and Core Target Genes, Survival Analyses for Hub Genes, Experimental validation	In HCC, ESR1 and CDK1 were both predicted as hub genes and targets of PADP.
Su et al. (2023)	GSE101685 GSE62232 GSE46408 GSE45627	Differential Gene expression analysis, PPI network analysis, Validation and survival analysis, MiRNA-Hub gene network construction, Drug-gene interaction analysis	CDK1 was predicted as one of the hub genes and 32 potential therapeutic targeting drugs for hub genes in HCC.
Moghimi et al. (2024)	GSE45267	Differential gene expression analysis, PPI network and modular analysis, Analysis of gene-disease association, Prediction of Pharmacological targets	CDK1 and ESR1 were among the significant DEGs in HCC. However, they were not among the hub genes upon further analysis
Ren and Feng (2024)	GSE84402 GSE12148 E-GEOD-19665	Differential gene expression analysis, PPI network analysis, Hub gene validation and survival analysis. Drug-target interaction analysis	CDK1 was associated with poor prognosis in HBV associated with HCC. Identified 6 drugs associated with CDK1
Ghosh et al., 2025 (present study)	GSE41804 GSE45267 GSE62232 GSE112790 GSE121248	Differential gene expression analysis, PPI network analysis and hub gene selection, gene selection based on Transcription Factor and miRNA targets, Survival analysis and validation of hub genes, Virtual screening ADMET analysis, molecular docking, Molecular Dynamics Simulation	Our bioinformatics approach, involving transcriptomics data analysis predicted CDK1 and ESR1 as hub genes associated with HCC as well as further exploration with computational drug discovery methods predicted Digoxin as a promising candidate for CDK1/CKS2 inhibition

A comprehensive study of the chemical drug-likeness of the seven drugs obtained through virtual screening, detailed ADME analysis and toxicity calculations. These parameters were instrumental in ranking the selected drugs for further docking and simulation studies, ultimately guiding the prediction of potential candidates. Key assessments include the PAMPA assay, which measures drug permeability and passive diffusion across an artificial membrane (Di et al., 2003). This metric, combined with Caco2 permeability, provides insights into the absorption and distribution of drugs within critical human physiological areas, such as the gastrointestinal tract, olfactory epithelium, blood-brain barrier (BBB), and nasal mucosa. The role of P-glycoproteins further complicates this landscape; these proteins can either enhance drug efficacy by transporting molecules or inhibit their action as Pgp inhibitors, ultimately influencing therapeutic outcomes (Wu et al., 2023; Wang et al., 2019).

In addition to these parameters, plasma protein binding (PPB) plays a crucial role in drug distribution after administration. This aspect assesses how compounds interact with plasma proteins like serum albumin and glycoproteins, significantly affecting their bioavailability and therapeutic effectiveness (Li et al., 2022). The BBB parameter also indicates the likelihood that a drug can successfully penetrate the central nervous system (CNS) through endothelial cells, highlighting its importance in neuropharmacology (Cheng et al., 2012; Daneman and Prat, 2015). Furthermore, cardiac rhythm regulation, influenced by hERG blockers, which control potassium ion channels in the heart, presents another layer of consideration for drug safety (Choi et al., 2020). This parameter, along with assessments of carcinogenicity, eye corrosion, irritation, hepatotoxicity, and neurotoxicity, provides a comprehensive view of the potential risks associated with each drug candidate.

Building on a comprehensive study of the chemical drug-likeness of selected drugs, molecular docking studies present additional challenges, particularly in determining the necessary flexibility of the receptor backbone. This flexibility is crucial for accurately predicting the interactions of ligands with their targets. Although several methods exist to address this issue, they often fall short with certain types of molecules (Meng et al., 2011). To overcome this challenge, the HDOCK server was used for docking analysis. This platform predicts various conformers of the same ligand and assesses their binding potential with the active site(s) of the receptor molecule, thereby reducing computational costs while still delivering reliable results (Martí-Renom et al., 2000). Docking scores were derived from a knowledge-based iterative scoring function, ITScorePP, which facilitates the effective ranking of conformers (Sievers et al., 2011; Remmert et al., 2012). The whole CDK1/CKS2 surface and the residues adjacent to the binding pockets of the ligands show polarity around either +0.1 or −0.1 charge (Supplementary Figures S1, S2). Intra-molecular interactions among the chains of CDK1 and CKS2 show a high number of salt bridges, hydrogen bonds and hydrophobic interactions (Supplementary Table S4; Supplementary Figure S3). This makes the protein somewhat rigid and may explain the phenomenon of no physical change inside the protein before and after binding with the ligands (Gunasekaran and Nussinov, 2007). Additionally, the final conformer structures were aligned with the initial model to compute the ligand root mean square deviation (RMSD).

Based on comprehensive ADMET analysis and docking studies, we identified Digoxin (DB00390) as the most promising candidate for further investigation. Digoxin has demonstrated anticancer activity against various human cancers. In the BALB/c nude mouse xenograft model, it inhibits lung cancer by inhibiting both DNA DSB and SSB repairs (Wang et al., 2020). Similarly, it shows its potential in arresting cervical cancer cells (Gan et al., 2020). Earlier researchers showed that various cardiac glycosides, including Digoxin and digitoxin, increased toxicity against melanoma cells as compared to normal human melanocytes and umbilical cord blood cells (Eskiocak et al., 2016). There have been reports in the last few years of clinical studies of Digoxin as an anticancer medicine, either by itself or with other chemotherapy drugs (Menger et al., 2013; Frankel et al., 2017; Huang et al., 2018). But researchers still don’t know exactly how Digoxin works to fight cancer. Very recently, Kangra et al. reported that Digoxin has been explored in 27 clinical trials so far, reflecting ongoing interest in its potential beyond heart conditions (Kangra et al., 2025). Of these, 11 have been completed, 7 are currently recruiting, and a few are in early or uncertain stages. Some trials were even terminated or withdrawn. These studies have tested Digoxin either alone or in combination with drugs like enzalutamide, rosuvastatin, capecitabine, lapatinib, metformin, and simvastatin. They span a range of cancer types - including prostate, breast, pancreatic, lung, and head and neck cancers - as well as solid tumors and neoplasms.

To learn more about Digoxin - CDK1 interaction, we performed subsequent simulations and evaluated the stability of the drug-receptor complex. Molecular dynamics simulations provide a detailed understanding of the receptor-ligand complex stability by examining various parameters, including RMSD, root mean square fluctuation (RMSF), radius of gyration (RoG), solvent-accessible surface area (SASA), MM-PBSA and various characteristics of the generated Hydrogen bonds between the protein and ligand. The study was carefully designed to address specific questions, ensure robust simulation setups, and thoroughly analyze the results (Hollingsworth and Dror, 2018). The findings demonstrated that the CDK1/CKS2-Digoxin complex maintained a stable conformation throughout the 100 ns simulation, with only minor instabilities and fluctuations occurring intermittently. These brief episodes of instability did not compromise the overall integrity of the complex, which remained compact until the simulation was finished.

In this study, we emphasize several aspects that contribute to the novelty and potential translational value of our findings. First, the integration of multiple, well-curated datasets enabled a robust and comprehensive analysis, strengthening the reliability of the identified candidate genes (Table 8). Second, we employed a target prioritization strategy based on regulatory complexity, selecting genes that are regulated by the highest number of transcription factors and miRNAs. Notably, genes targeted by multiple miRNAs were considered particularly promising, as such regulation often reflects their centrality and importance in disease-related biological pathways (Okada et al., 2016). Furthermore, the identification of Digoxin as a potential repurposable drug through computational analysis demonstrates the effectiveness of our method in revealing novel drug-disease connections. Although our *in silico* findings suggest that Digoxin may inhibit liver cancer growth via CDK1 regulation, its therapeutic use remains limited due to several factors. Firstly, Digoxin has a narrow therapeutic index, making dosing challenging without risking cardiotoxicity. Its known systemic toxicity, notably cardiac side effects, raises serious safety concerns. Furthermore, poor cancer cell selectivity and limited tumor-specific delivery impede successful targeting. Lastly, the lack of robust *in vivo* and clinical data in oncology restricts its immediate translational potential. To better understand how Digoxin might help treat liver cancer, future research should include more experimental repetitions and make use of advanced tools - like multi-omics data and clinical sample analysis. Studies such as Western blotting, nuclear/cytoplasmic fraction, immunofluorescence, and FACS (Lu et al., 2025), as well as *in-vivo* and *ex-vivo* experiments (Kurzeder et al., 2025), may be designed to validate these computational predictions and further explore the therapeutic potential of Digoxin in liver cancer. Overall, our *in silico* study demonstrates Digoxin’s strong impact on the CDK1/CSK2 complex and offers important insights for the development of more effective and selective therapies against liver cancer. These approaches can uncover the precise mechanisms behind Digoxin’s action and provide clearer insights into its potential as a safe and effective cancer therapy.

## 5 Conclusion

In conclusion, identifying reliable biomarkers is crucial for early diagnosis, prognosis, and effective treatment of hepatocellular carcinoma (HCC). By analyzing five microarray datasets, we identified 19 upregulated and 64 downregulated genes associated with HCC. Gene Ontology (GO) and KEGG pathway analyses revealed that downregulated genes play a significant role in HCC pathology. Notably, CDK1/CKS2 and ESR1 emerged as hub genes that correlated with poor overall and disease-free survival in patients, underscoring their potential as biomarkers for HCC. We further investigated CDK1/CKS2 using molecular docking and dynamic simulations to explore its therapeutic potential. Additionally, our study identifies Digoxin (DB00390) as a promising repurposed drug candidate to overcome the limitations of existing drugs. Overall, this study contributes to a deeper understanding of HCC biology and drug interactions, paving the way for improved therapeutic strategies.

## Data Availability

The original contributions presented in the study are included in the article/Supplementary Material, further inquiries can be directed to the corresponding authors.
