# Supplementary material for: Identification and therapeutic investigation of biomarker genes underpinning hepatocellular carcinoma: an in silico study utilising molecular docking and dynamics simulation
Source: Front Bioinform. 2025 Sep 19;5:1567748. doi: 10.3389/fbinf.2025.1567748 (PMC12491263; doi:10.3389/fbinf.2025.1567748)
Supplement: Supplementary file 4 [file DataSheet1.docx]

**Supplementary File 2**

The whole of CDK1/CKS2 surface and the residues adjacent to the binding pockets of the ligands shows polarity ranging from +0.1 to -0.1 charge. This makes the protein somewhat rigid and may explain the phenomenon of no physical change inside the protein before and after binding with the ligands (Gunasekaran et al, 2007).


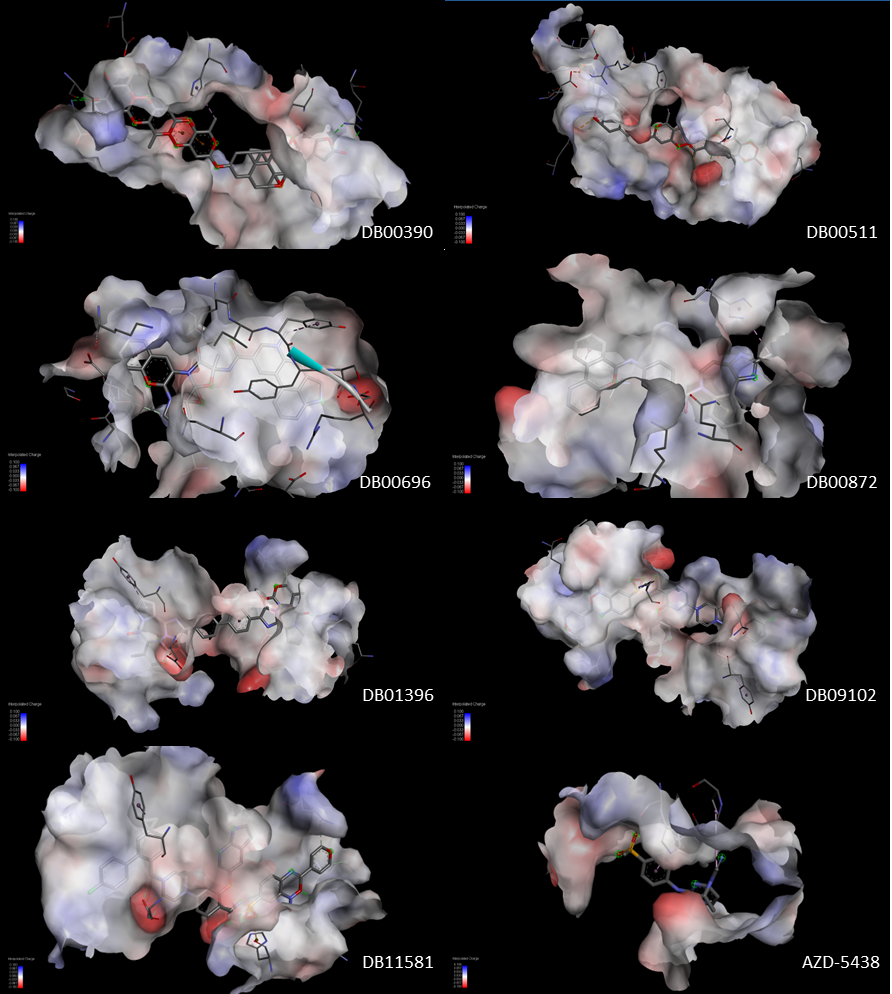


**Supplementary Figure S1:** The polarity and charge distribution in the residues of CDK1/CKS2 interacting with various drugs. charge varies from +0.100 (blue) to -0.100 (red)


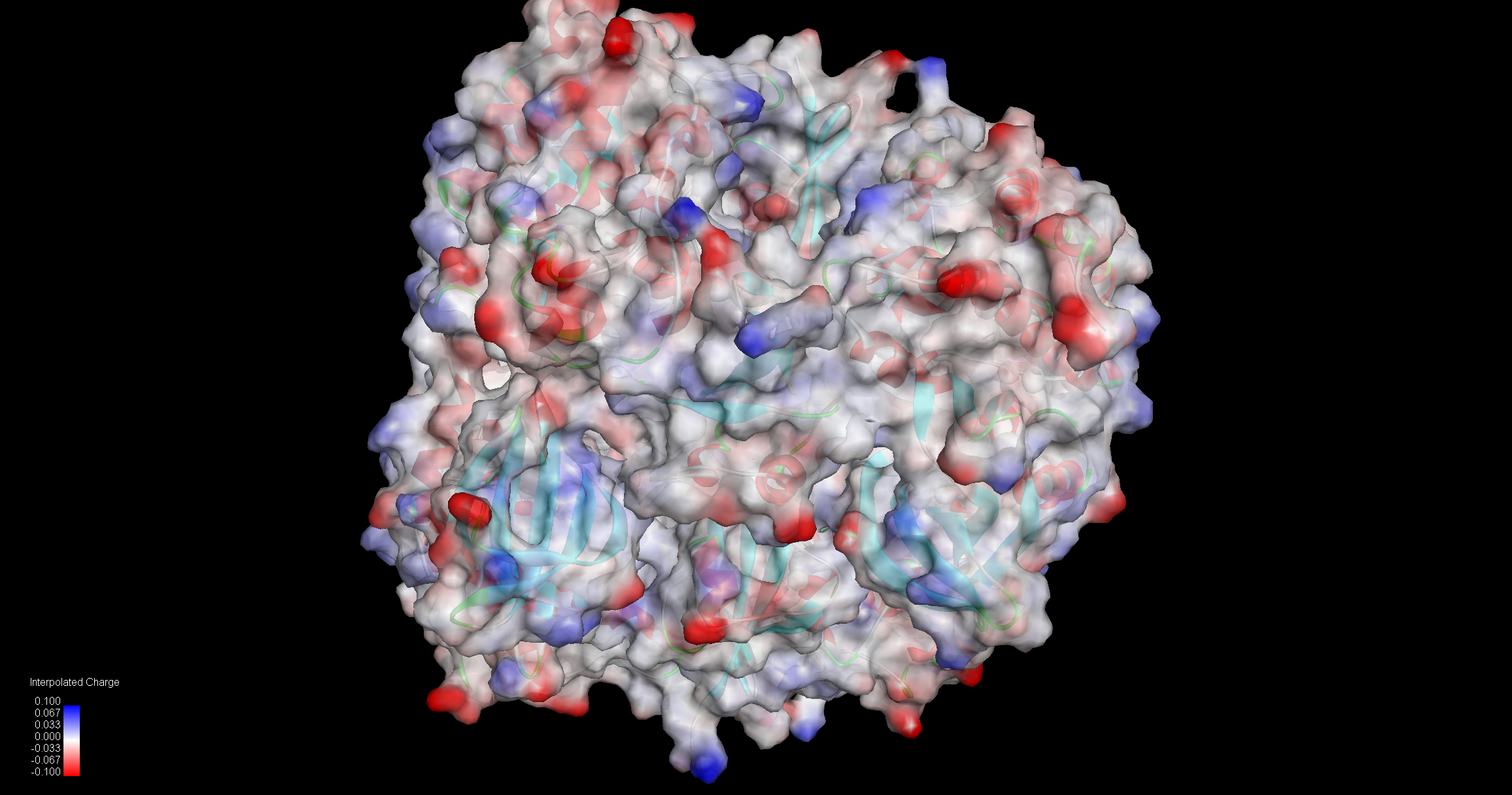


**Supplementary Figure S2:** The polarity and charge distribution in the residues of CDK1/CKS2

Reference:

Gunasekaran, K., & Nussinov, R. (2007). How different are structurally flexible and rigid binding sites? Sequence and structural features discriminating proteins that do and do not undergo conformational change upon ligand binding. *Journal of molecular biology*, *365*(1), 257-273.

Protein chain A, C, E, G belongs to CDK1 and B, D, F, H makes the CKS2 part of the whole complex. The intra molecular interactions i.e., salt bridges, hydrogen bonds and hydrophobic interactions are shown in the table below.

| CDK1 chain ID | CKS2 chain ID | No. of Salt Bridge | No. of H-bond | No. of Hydrophobic interactions |
| --- | --- | --- | --- | --- |
| A | B | 1 | 6 | 14 |
| A | E | 3 | 11 | 10 |
| A | F | 1 | 3 | 7 |
| A | H | 1 | 6 | 8 |
| B | D | 0 | 3 | 5 |
| B | G | 1 | 4 | 8 |
| C | D | 1 | 6 | 14 |
| C | F | 0 | 4 | 6 |
| C | G | 3 | 15 | 13 |
| C | H | 1 | 1 | 3 |
| D | E | 1 | 6 | 7 |
| D | G | 0 | 2 | 3 |
| E | F | 2 | 4 | 16 |
| F | H | 1 | 1 | 10 |
| G | H | 1 | 4 | 20 |

**Supplementary Table S4:** Number of salt bridges, hydrogen bonds and hydrophobic interactions found among the chains of CDK1 and CKS2.


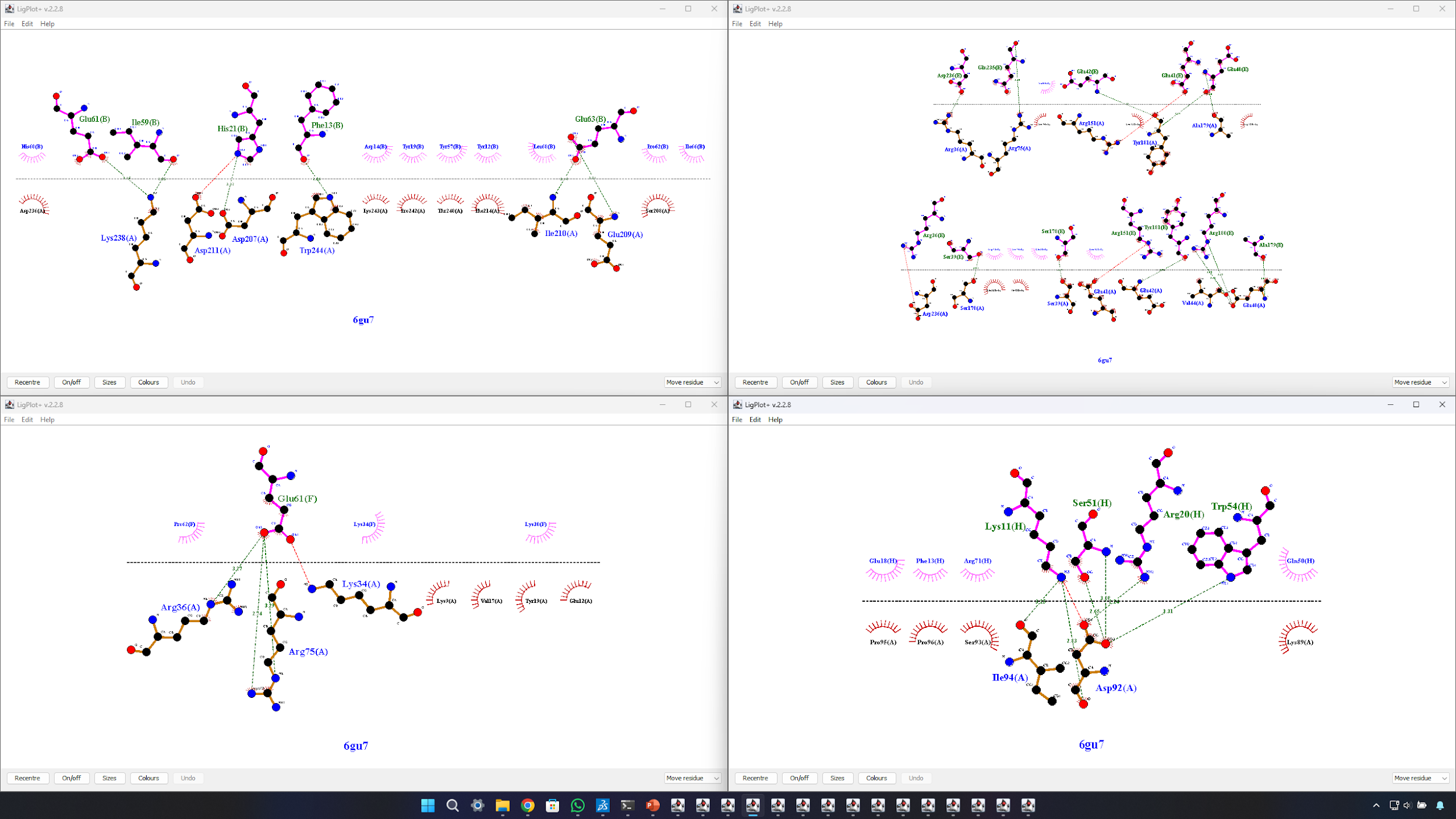


(a)


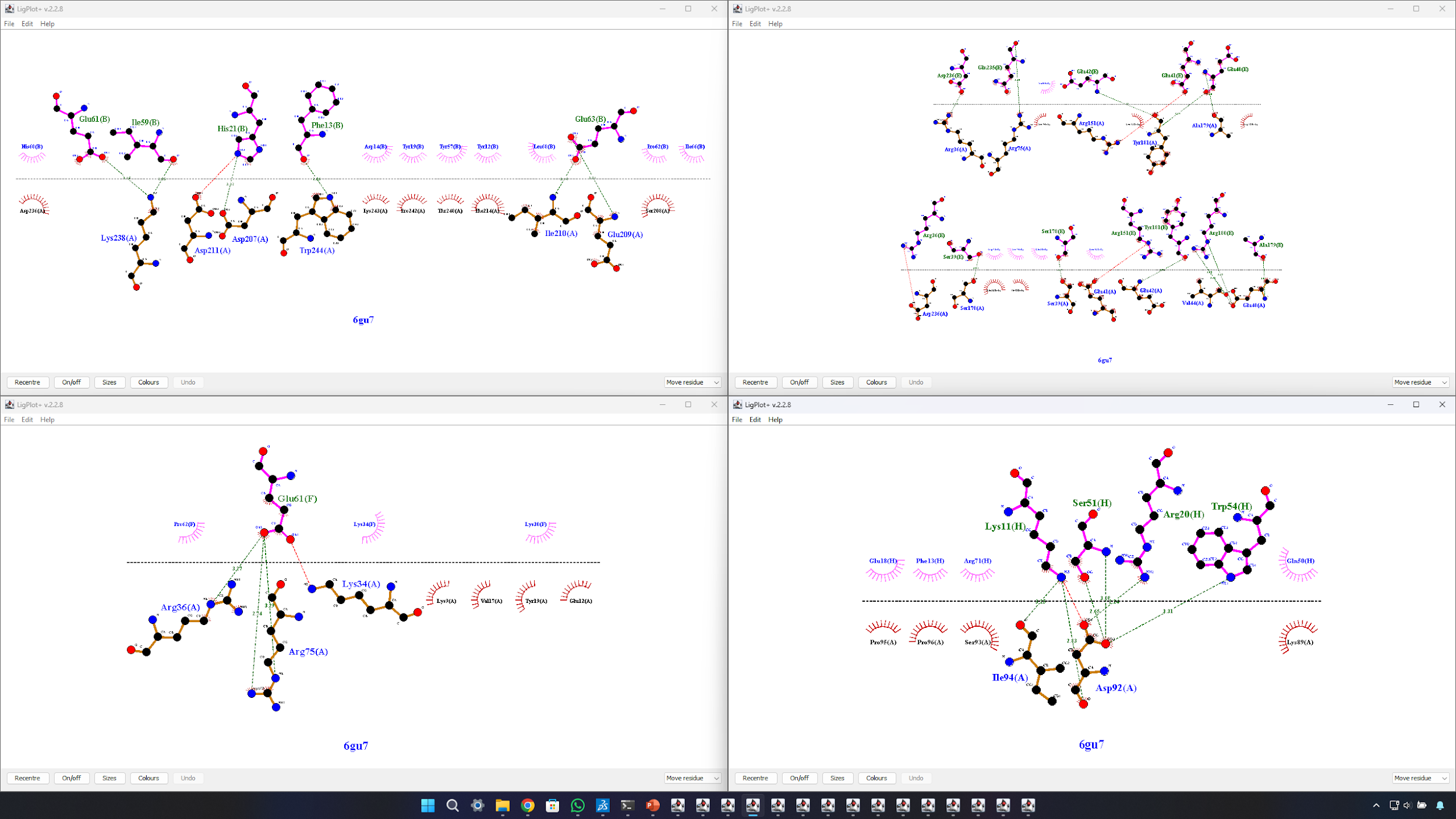


(b)


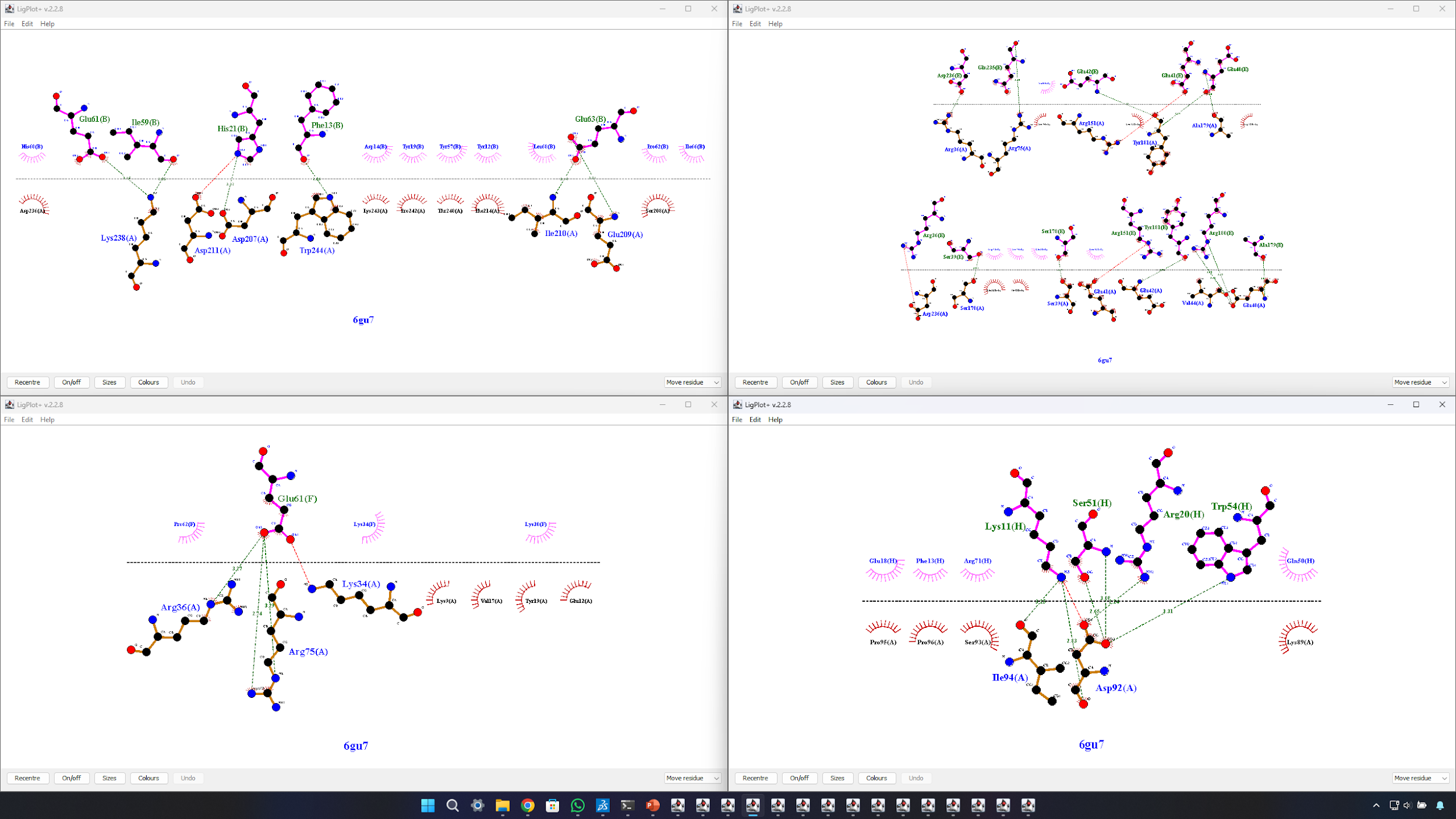


(c)


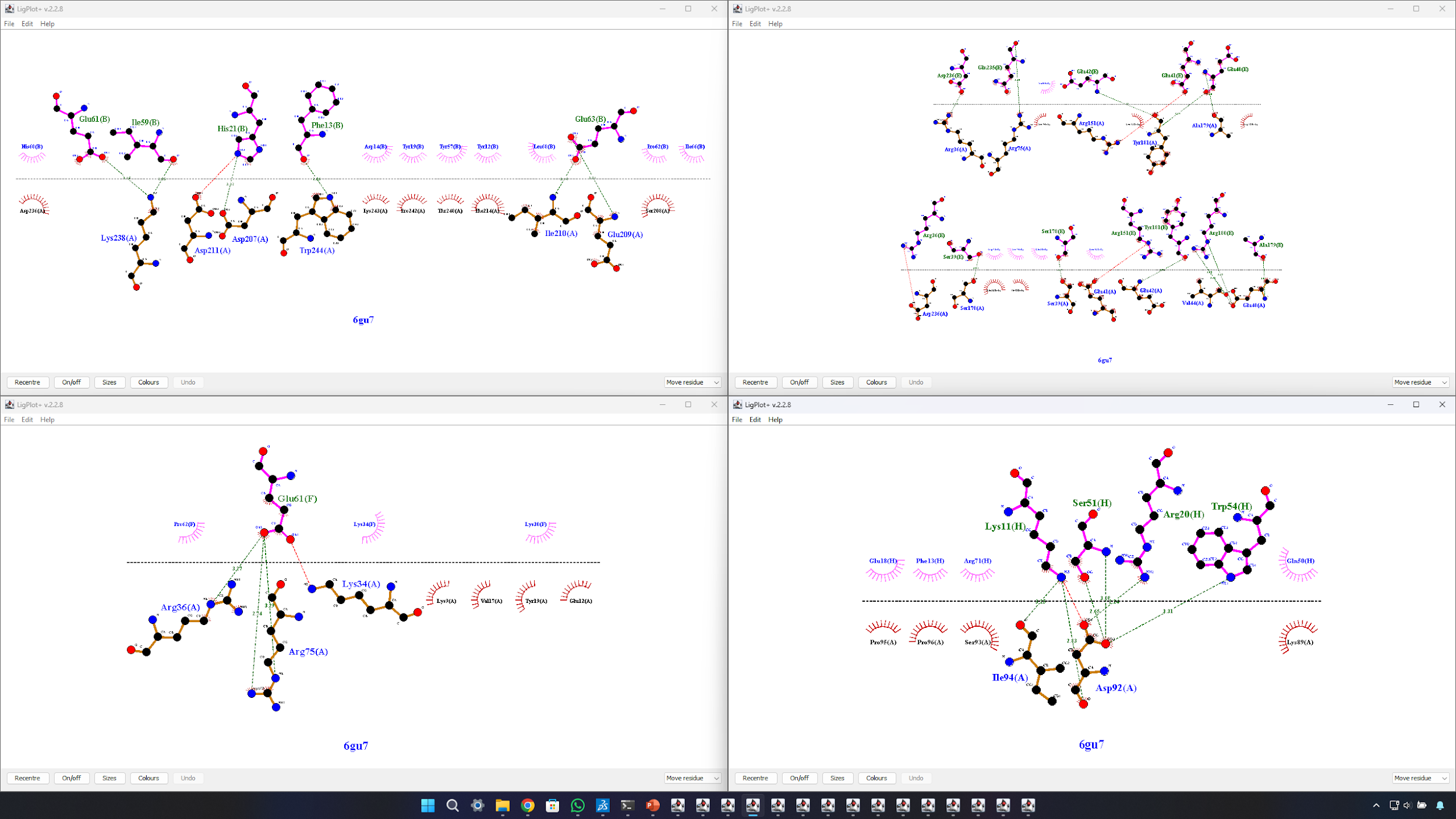


(d)


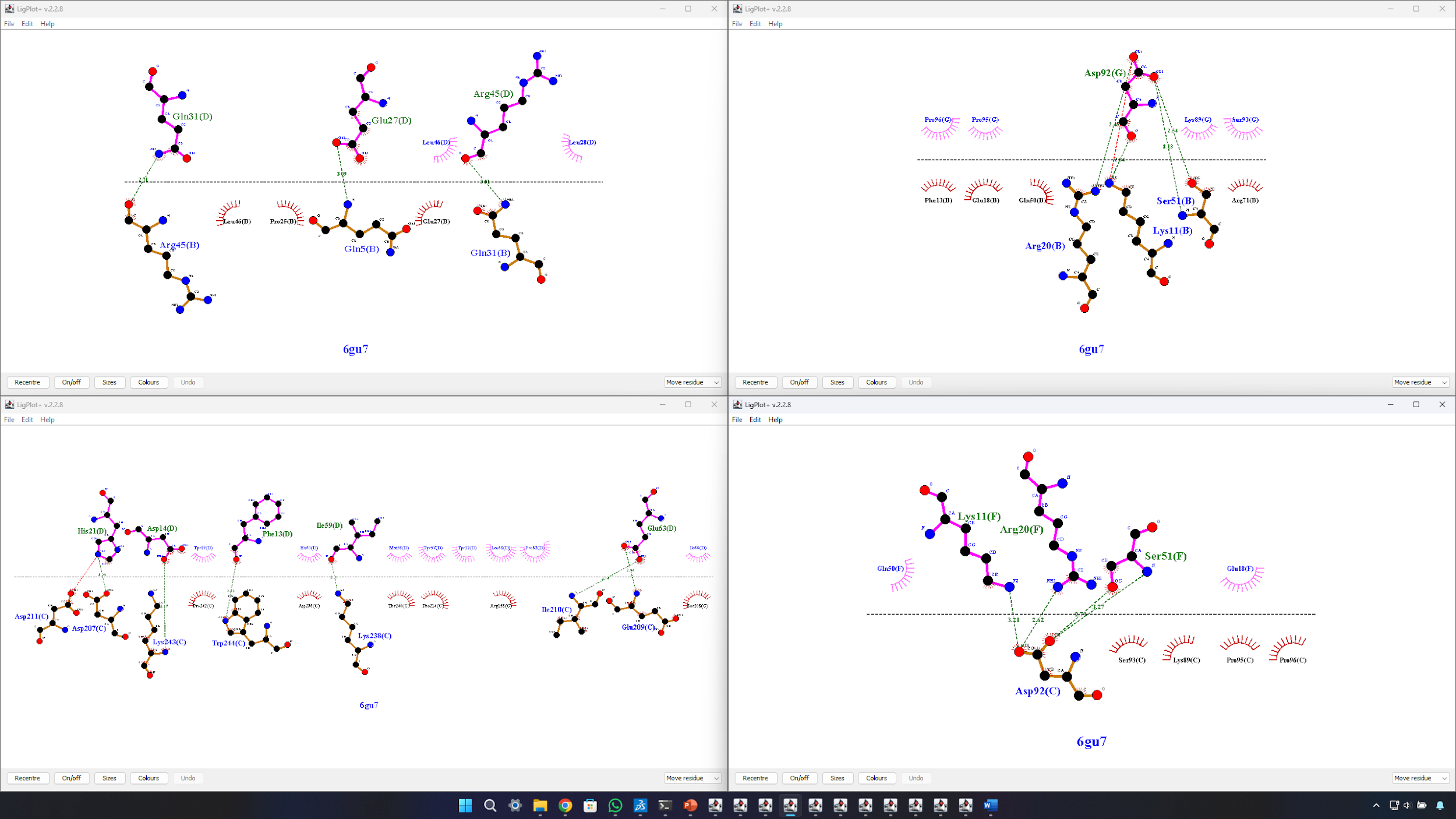


(e)


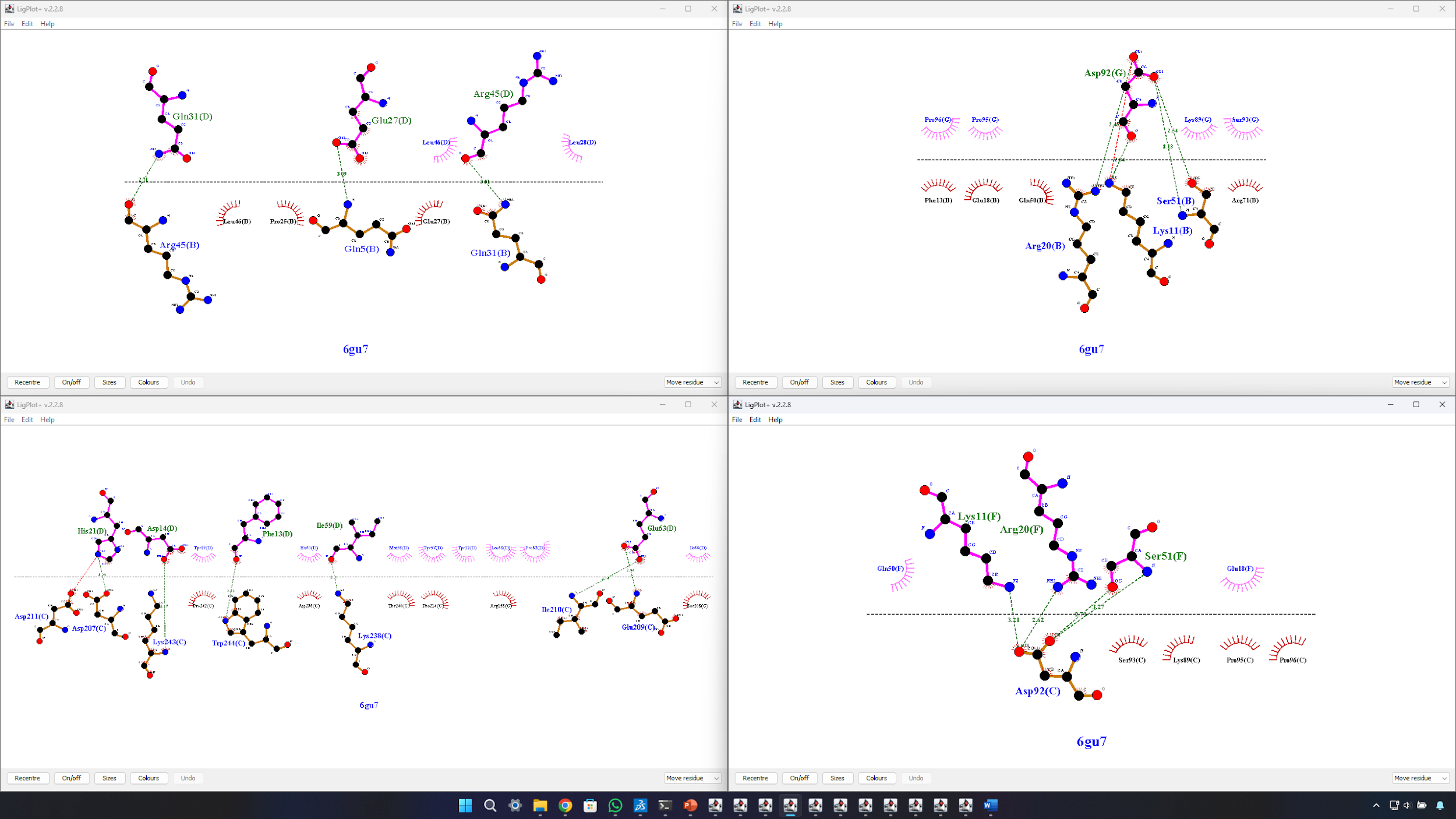


(f)


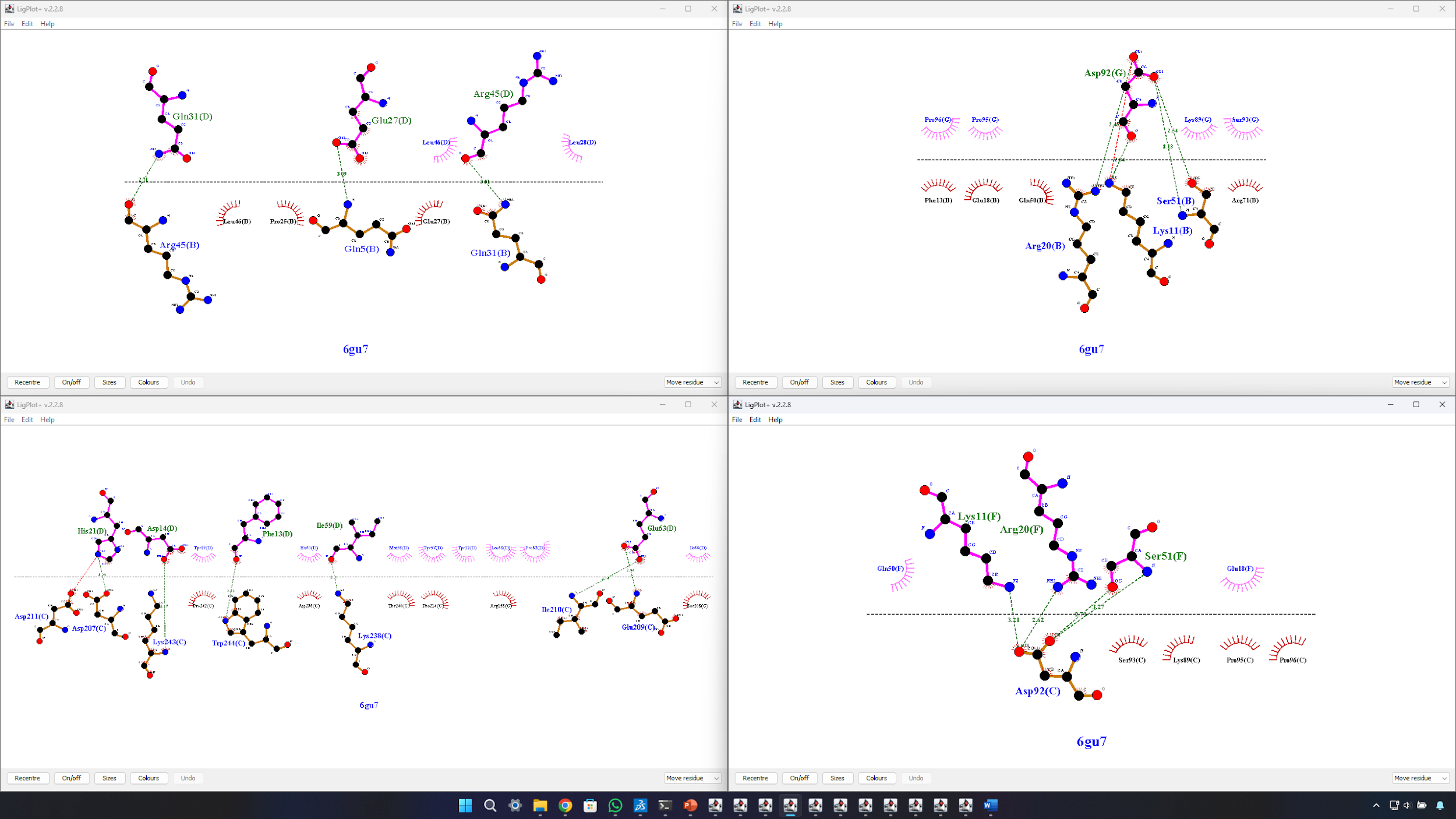


(g)


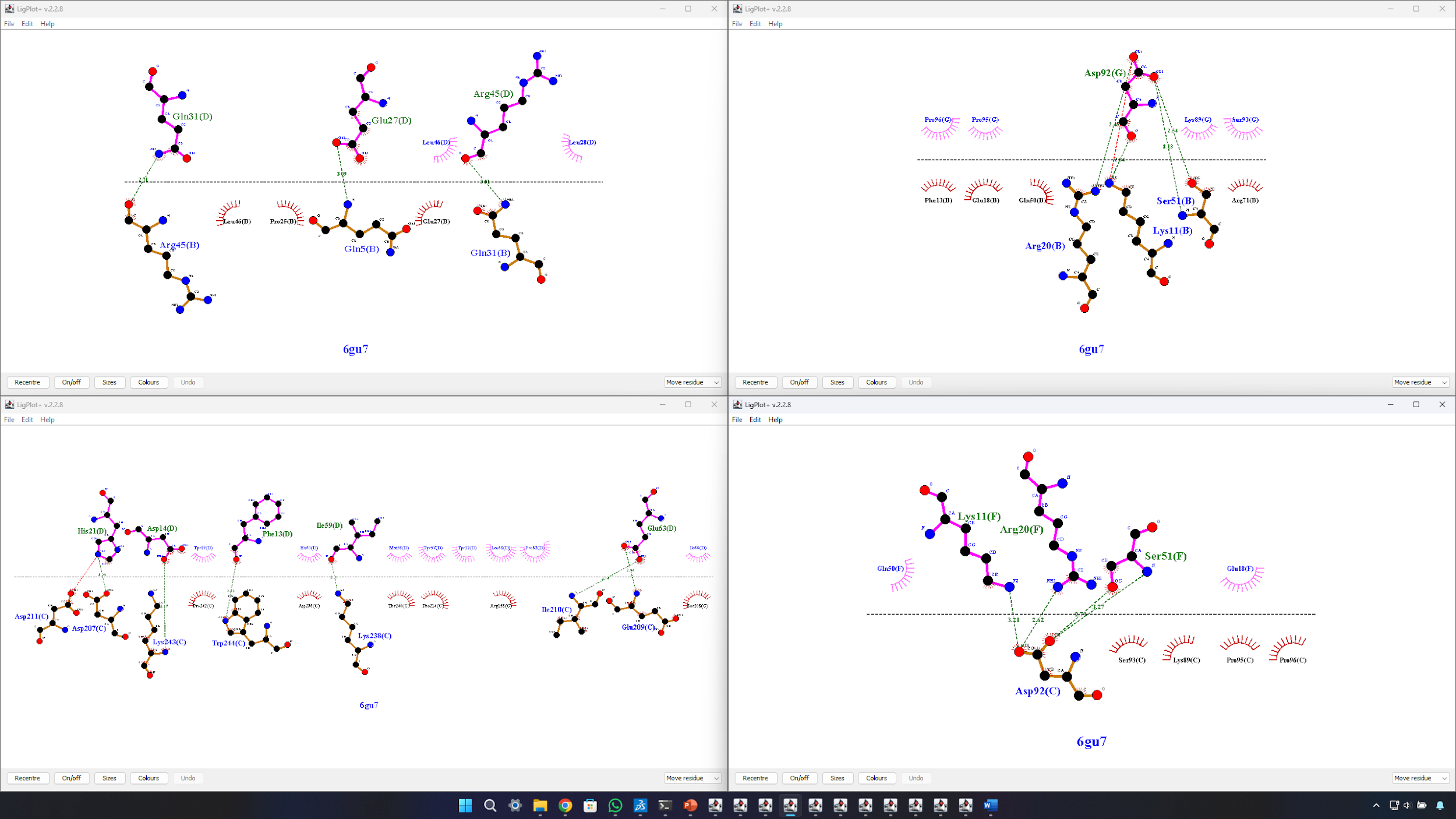


(h)


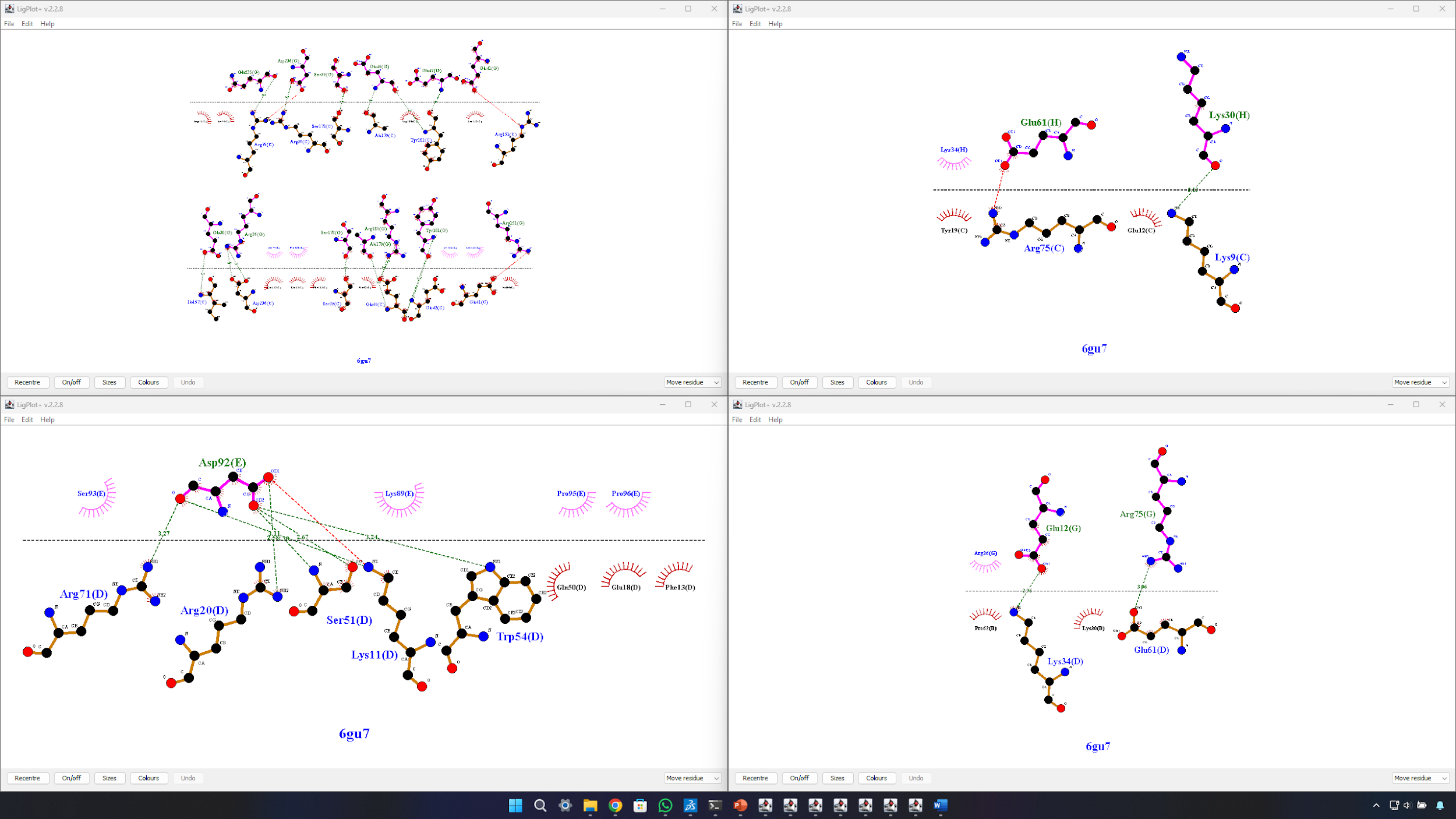


(i)


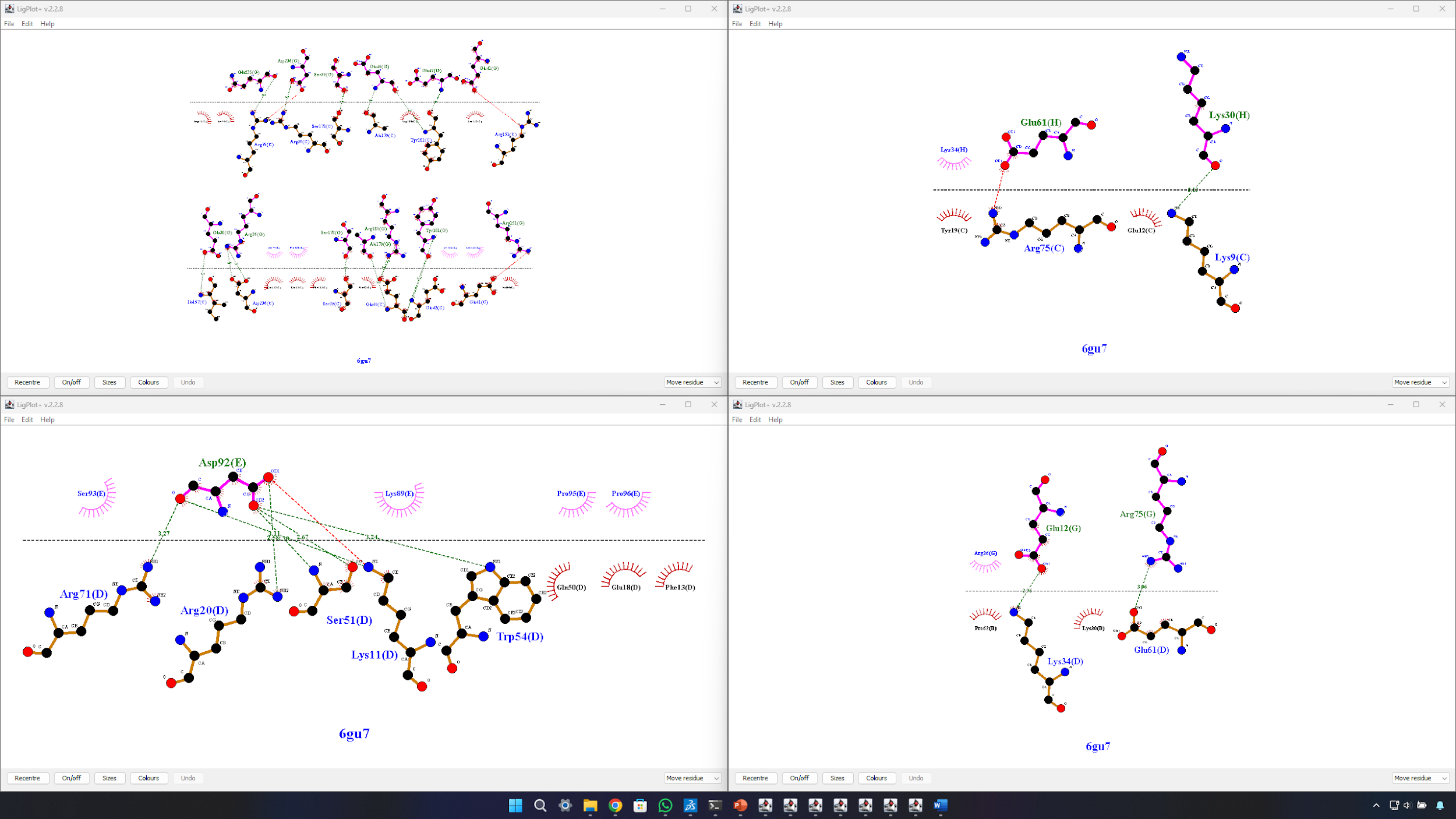


(j)


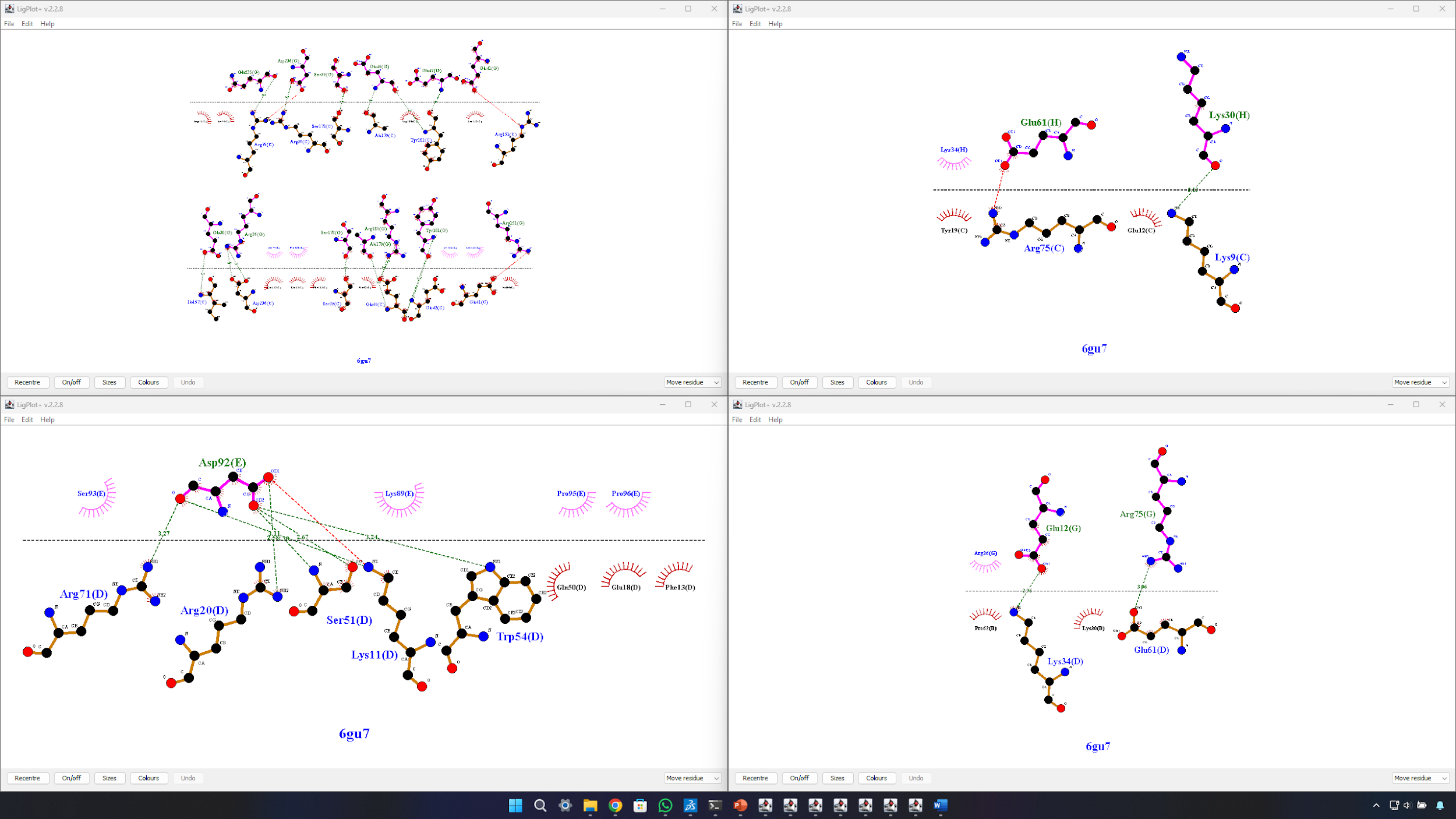


(k)


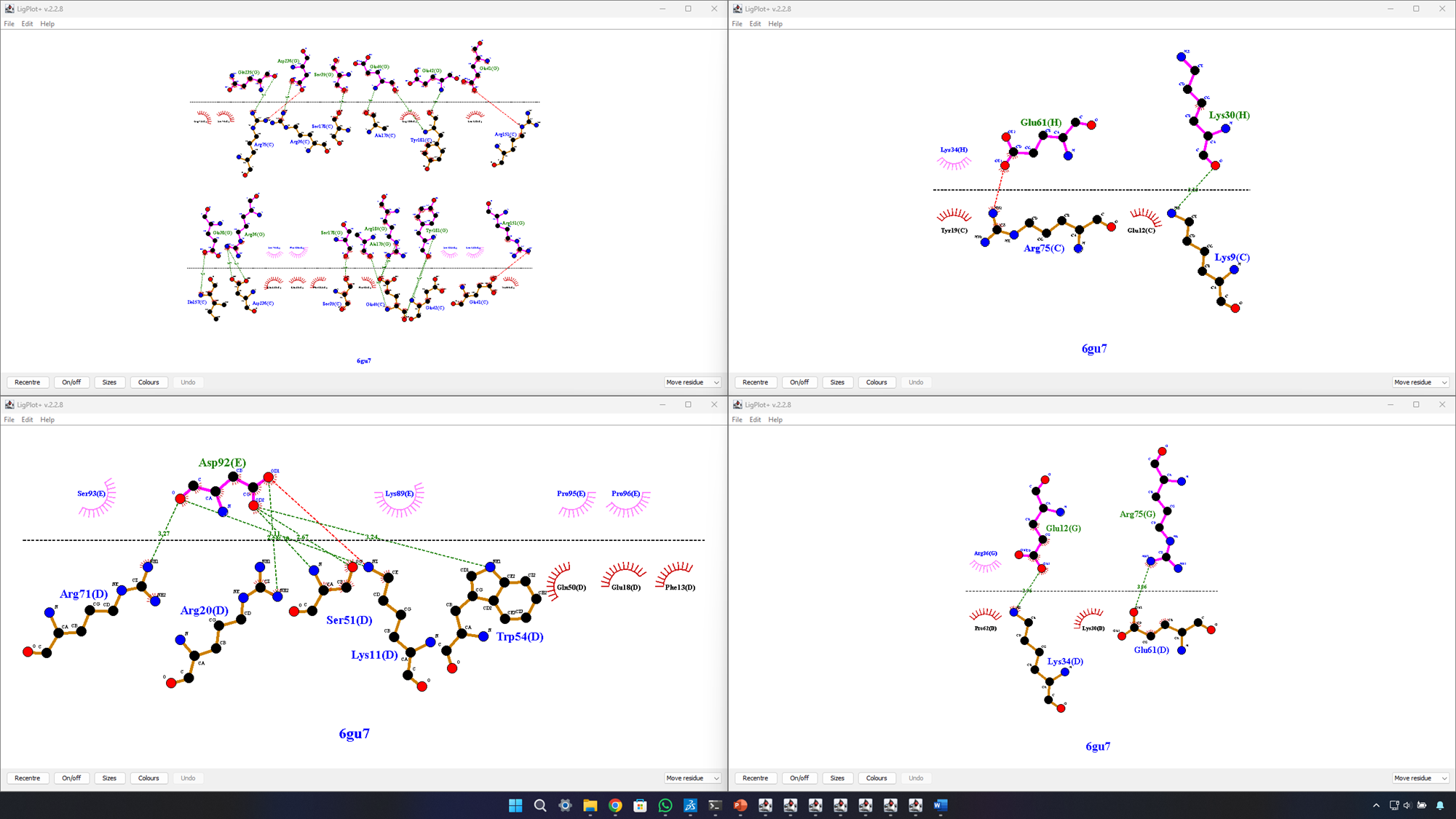


(l)


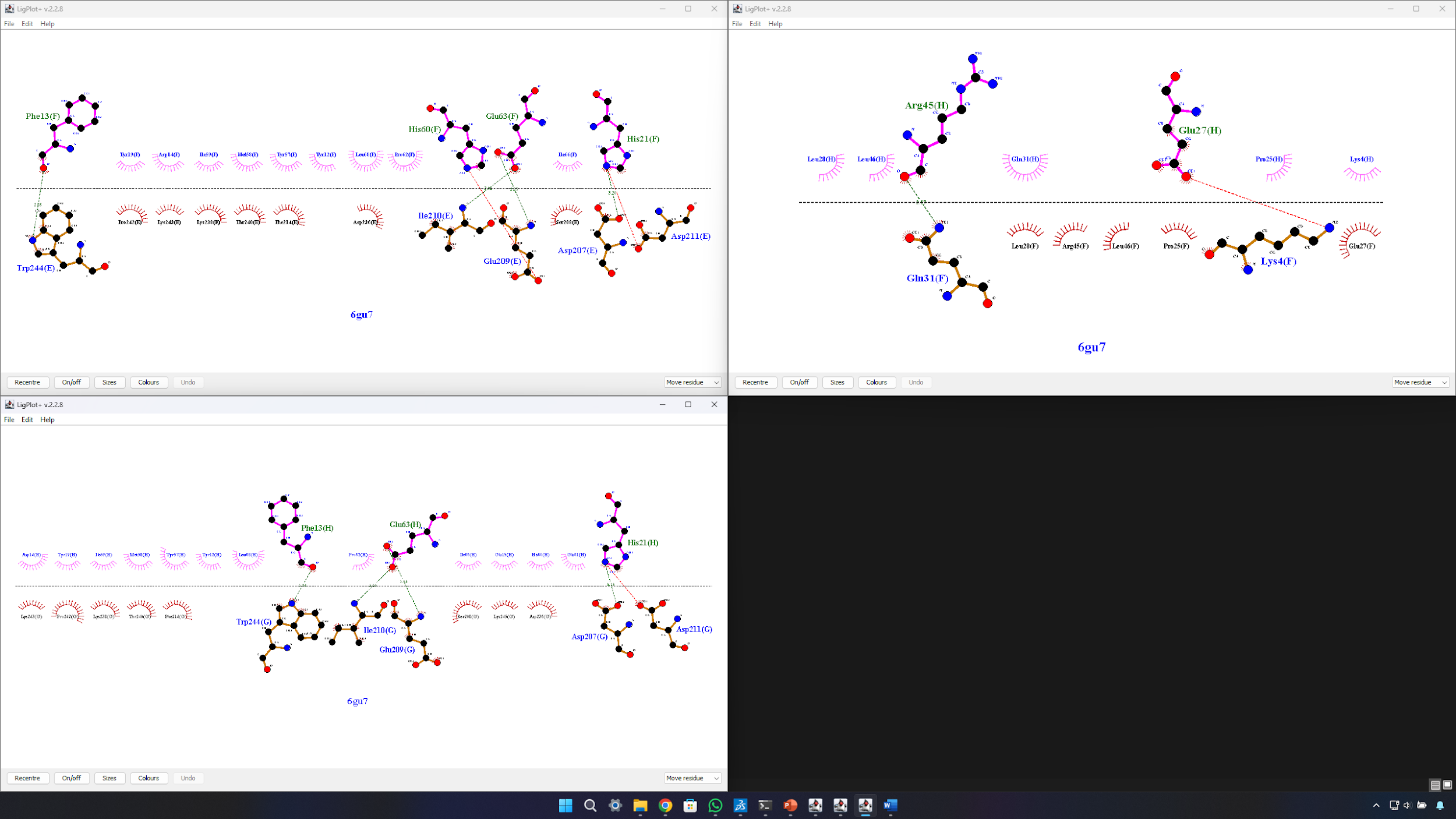


(m)


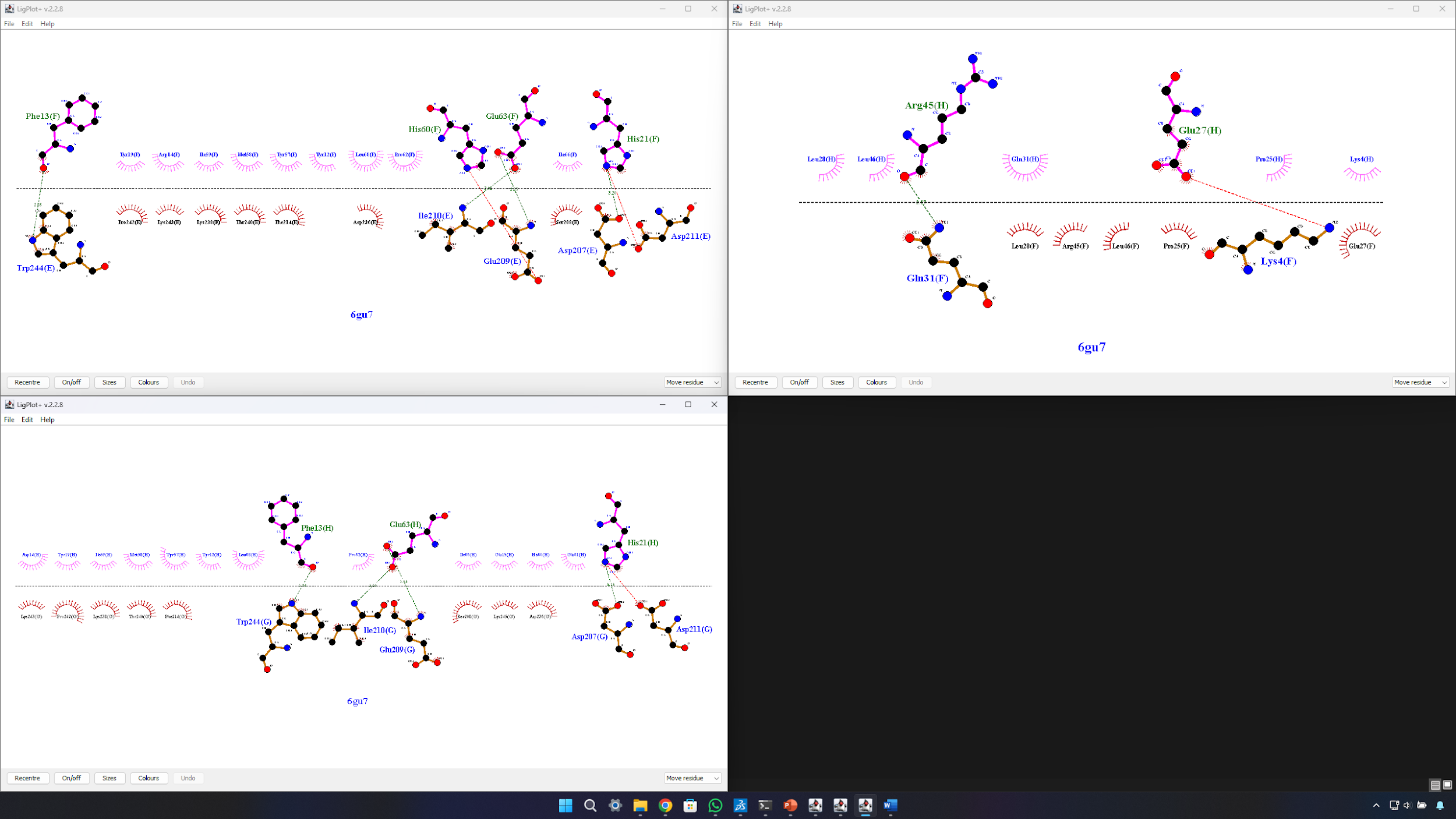


(n)


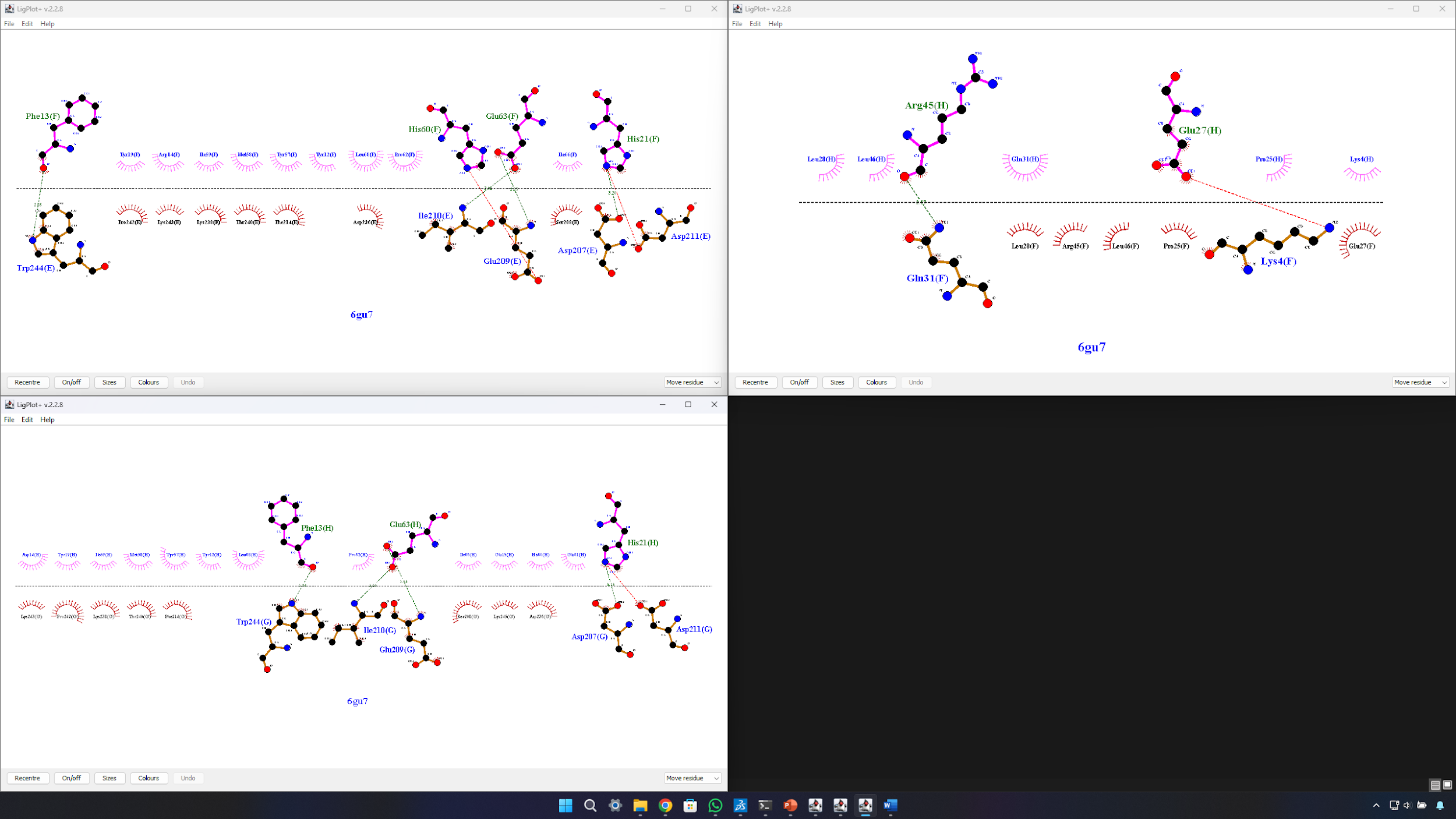


(o)

**Supplementary Figure S3**: Intra molecular interactions among the chains of CDK1 and CKS2 analyzed in Ligplus
